# Integrated Bioinformatics Analyses of *PIN1*, *CKX*, and Yield-Related Genes Reveals the Molecular Mechanisms for the Difference of Seed Number Per Pod Between Soybean and Cowpea

**DOI:** 10.3389/fpls.2021.749902

**Published:** 2021-11-29

**Authors:** Lü-Meng Liu, Han-Qing Zhang, Kun Cheng, Yuan-Ming Zhang

**Affiliations:** Crop Information Center, College of Plant Science and Technology, Huazhong Agricultural University, Wuhan, China

**Keywords:** seed number per pod, ovule number, *PIN1*, *CKX*, soybean, cowpea, interaction network, yield

## Abstract

There is limited advancement on seed number per pod (SNPP) in soybean breeding, resulting in low yield in China. To address this issue, we identified *PIN1* and *CKX* gene families that regulate SNPP in *Arabidopsis*, analyzed the differences of auxin and cytokinin pathways, and constructed interaction networks on *PIN1, CKX*, and yield-related genes in soybean and cowpea. First, the relative expression level (REL) of *PIN1* and the plasma membrane localization and phosphorylation levels of PIN1 protein were less in soybean than in cowpea, which make auxin transport efficiency lower in soybean, and its two interacted proteins might be involved in serine hydrolysis, so soybean has lower SNPP than cowpea. Then, the *CKX* gene family, along with its positive regulatory factor *ROCK1*, had higher REL and less miRNA regulation in soybean flowers than in cowpea ones. These lead to higher cytokinin degradation level, which further reduces the REL of *PIN1* and decreases soybean SNPP. We found that *VuACX4* had much higher REL than *GmACX4*, although the two genes essential in embryo development interact with the *CKX* gene family. Next, a tandem duplication experienced by legumes led to the differentiation of *CKX3* into *CKX3a* and *CKX3b*, in which *CKX3a* is a key gene affecting ovule number. Finally, in the yield-related gene networks, three cowpea *CBP* genes had higher RELs than two soybean *CBP* genes, low RELs of three soybean-specific *IPT* genes might lead to a decrease in cytokinin synthesis, and some negative and positive SNPP regulation were found, respectively, in soybean and cowpea. These networks may explain the SNPP difference in the two crops. We deduced that *ckx3a* or *ckx3a ckx6 ckx7* mutants, interfering *CYP88A*, and over-expressed *DELLA* increase SNPP in soybean. This study reveals the molecular mechanism for the SNPP difference in the two crops, and provides an important idea for increasing soybean yield.

## Introduction

Soybean is a major oil crop in China and an important source of plant protein for human beings. However, the soybean imports of China have increased from 58.38 million tons in 2012 to 100.32 million tons in 2020, which has seriously affected the food security in China. In order to revitalize the soybean industry in China, the key is to increase the yield per unit area. Although seed number per pod (SNPP) in soybean is an important yield component factor, such as the utilization of gene *ln* in Zhonghuang13 (Zhu et al., 2020), the advance in long-term soybean breeding is limited. Thus, increasing SNPP of soybean is a new direction for increasing its yield per unit area. Although elite genes for important traits in most crops frequently come from their wild or closely related species, and the SNPP of cultivated soybean is almost the same as that of wild soybean, cowpea has much higher SNPP (approximately 12) than soybean (2–4). Therefore, it is necessary to investigate the molecular mechanism of SNPP difference between soybean and cowpea.

In the past decade, efforts have been made to dissect the genetic foundation and molecular mechanism of SNPP. As described by Carlson and Lersten (2004), the SNPP difference is mainly caused by ovule number per pistil. Jeong et al. (2012) cloned a soybean locus *ln* and proved its pleiotropy for narrow-leaf and higher seed number. Cai et al. (2021) used CRISPR/Cas9 technology to edit gene *Ln* and its homologous genes in soybean cultivar “Huachun 6” to create a new material carrying gene *ln*, which is available for the future field breeding. To confirm whether the difference of SNPP between *Ln* and *ln* genotypes is caused by ovule number per pistil, Fang et al. (2013) found that ovate leaflet cultivar “Han 2296” with two-seeded pods has two to three ovules per ovary, and narrow leaflet cultivar “Lvbaoshi” with four-seeded pods has three to four ovules per ovary. This indicates that the *Ln* gene may influence SNPP by regulating ovule number per pistil. In *Arabidopsis thaliana*, ovules are produced from the placenta as lateral organs, and the ovule number in each ovary is regulated by plant hormones, such as auxin and cytokinin (Bencivenga et al., 2012; Galbiati et al., 2013; Reyes-Olalde et al., 2013; Cucinotta et al., 2014).

Auxin is required for placenta formation and ovule growth, and reduced auxin biosynthesis or transport in plants leads to severe defects in gynoecium development, resulting in loss of placenta tissue and ovule (Nemhauser et al., 2000; Nole-Wilson et al., 2010). The auxin exogenous vector PIN1 is one main element that regulates auxin accumulation at various ovule development stages. The PIN1-dependent auxin efflux mediates primordium development by supplying the apex of the ovule primordium with an auxin maximal zone (Benková et al., 2003; Ceccato et al., 2013). The expression level of *PIN1* in *Arabidopsis pin1-5* mutants and the number of ovules are reduced, compared with those in wild-type *Col-0* (Bencivenga et al., 2012). Yu et al. (2020) showed that ovule primordia initiate asynchronously and new ovule primordia formation still requires the auxin maximal zone. Taken together, it is of great significance to investigate the difference of PIN1-mediated auxin transport in order to dissect the molecular mechanism for the difference of ovule numbers between soybean and cowpea.

Cytokinins (CK) are positive regulators of shoot apical meristem (Werner et al., 2001, 2003; Riefler et al., 2006; Kurakawa et al., 2007) and play important roles in ovule development. The defects in plant cytokinin production or perception affected ovule formation (Higuchi et al., 2004; Nishimura et al., 2004; Bencivenga et al., 2012). After the treatment of synthetic cytokinin 6-benzylaminopurine (BAP) in *Arabidopsis thaliana* and *Brassica napus* inflorescence, the expression level of *PIN1* in the pistil and the ovule number per pistil increased (Bencivenga et al., 2012; Zuñiga-Mayo et al., 2018). Cytokinin is specifically degraded by Cytokinin dehydrogenase (CKX). As compared to wild types, *ckx3 ckx5* mutants in *Arabidopsis* increased SNPP and seed yield (Bartrina et al., 2011), and *bnckx3 bnckx5* sixfold mutant in *Brassica napus* increased ovule numbers per pistil and pod numbers, resulting in an increase in final seed yield (Schwarz et al., 2020). In conclusion, CKX-mediated cytokinin degradation may be the key to improving crop yield.

To dissect possible molecular mechanisms for the SNPP difference between soybean and cowpea, first, in this study, we identified *PIN1* and *CKX* gene families in soybean and cowpea genomes, and analyzed the differences of auxin and cytokinin pathways between the two crops to mine SNPP-related genes. Then, we constructed interaction networks on *PIN1*, *CKX*, SNPP, and yield-related genes in soybean and cowpea to explore possible molecular mechanisms for the SNPP difference in the two legumes. In addition, we discussed how to improve SNPP in soybean.

## Materials and Methods

### Data Sources

The whole genome protein sequences in *Arabidopsis* and soybean were downloaded from TAIR database (^1^ TAIR release 10) and SoyBase database (^2^ version wm82.a2. V1; Schmutz et al., 2010), respectively, while the whole genome protein sequences in cowpea and kidney bean were download from Legumeinfo database (^3^ Lonardi et al., 2019; version gnm1.ann1).

Transcriptome datasets were downloaded from Phytozome database^4^ for soybean flower and embryo, Legumeinfo database (see text footnote 3) for cowpea and kidney bean flower and embryo, NCBI^5^ for soybean seed, and NCBI^6^ for cowpea seed.

Seven hundred fifty-six mature miRNA sequences in soybean were downloaded from miRBase database^7^, while 656 mature miRNA sequences in cowpea were downloaded from Martins et al. (2020).

### Identification of Homologous Gene Families and Phylogenetic Tree Construction

The OrthoFinder was used to identify homologous gene families (Emms and Kelly, 2015) when we input the whole genome protein sequence for *Arabidopsis*, soybean, and cowpea, and adopted the default parameters. Based on the homologous gene families, the number of unique, single-copy, multi-copy, and unclustered gene families and protein numbers in soybean and cowpea were counted.

The phylogenetic tree was constructed by Neighbor–Joining (NJ) approach using the MEGA7 software^8^ and protein sequence alignment was carried out by the ClustalW method. The number of replicates in Bootstrap Test was set as 1,000, and the other parameters were set as default values. The final result tree file was visualized and beautified using the ITOL online tool^9^.

### Gene Structure and Protein Structure Analyses and Subcellular Localization Prediction

The online tools GSDS2.0 (^10^ Gene Structure Display Server), pfam^11^, Wolf PSORT^12^, and MEME^13^ were used to graphically display gene structure, search the target protein sequences of conservative domain structure, predict the subcellular localization of target protein, and analyze conservative motif, respectively.

### Collinearity and Protein Interaction Analyses

The collinearity among *Arabidopsis thaliana*, soybean, cowpea, and the collinearity within soybean species, were analyzed using TBtools software (Chen et al., 2020). The sequences of target proteins in *Arabidopsis* were submitted to the STRING database^14^ to search for interaction proteins with experimental evidence, active interaction sources in soybean were set up as “all,” and combined score >0.4 was regarded as the cut-off point of significance. In cowpea, there is no interacted protein database available. Thus, interaction protein network in mung bean (*Vigna radiata*), which is the closest species of cowpea, was used to represent the one in cowpea. Here, comparative genomics analysis between cowpea and mung bean was conducted on Phytozome and OrthoFinder. The protein interaction network was beautified by the Cytoscape software. Gene function annotation was conducted on SoyBase and Phytozome for soybean and TAIR for *Arabidopsis*.

### Expression Pattern Analysis and Relative Expression Level Comparison

The expression patterns of genes in flower, leaf, stem, root, pod, and seed in soybean, cowpea, and kidney bean were analyzed via standardized expression levels, log_2_(*y* + 1), where *y* was real expression level. Relative expression levels were defined as the ratio of the expression level of one gene to average expression level of all the genes in the species (deleting the genes with expression level less than 1.0) (Zhang et al., 2018; Zhou et al., 2019; Cheng et al., 2021) and used to compare the differences of expression levels of genes in the flowers of soybean and cowpea. The TBtools and SigmaPlot software packages were used to draw HeatMap and relative expression levels, respectively.

### Prediction of MicroRNAs and Expansion Pattern Analysis of Gene Families

The online tool psRNATarget^15^, a plant small RNA target analysis server, was used to predict micoRNAs, implemented by Schema V2 (2017 release) where expected value was set as 4 and other parameters were set as their default values. The number of predicted miRNA for each gene was counted and then plotted by SigmaPlot.

In this study, we focused on two types of patterns in gene expansion: tandem and segmental duplications. The above collinearity results were used to determine duplicated gene pairs, and these gene pairs were compared with the gene pairs downloaded from PlantDGD^16^ to validate the predicted gene pairs. The formula T = Ks/2λ was used to calculate the date of occurrence of repeated events, where λ is equal to 6.1 × 10^–9^ (Lynch and Conery, 2000).

## Results

### Identification and Copy Number Analysis of Homologous Gene Families in *Arabidopsis*, Soybean, and Cowpea

To identify orthologous genes (OGs) in soybean and cowpea, all the genes in *Arabidopsis*, soybean, and cowpea were clustered using the OrthoFinder software. As a consequence, 113,233 protein-coding genes from the three species were clustered into 21,582 OGs (Supplementary Table 1), with each OG representing a gene family. Among these gene families, 1,541 (7.14%) were identified as soybean-specific gene families, and only 451 (2.09%) were identified as cowpea-specific gene families. The two proportions (9.23%) were very low, indicating the very high similarity in the evolutionary process between soybean and cowpea. Meanwhile, 549 (2.54%) OGs with single-copy soybean and multi-copy cowpea genes was significantly lower than 10,196 (47.24%) OGs with single-copy cowpea and multi-copy soybean genes. This means that soybean underwent a unique allotetraploidy event.

### Difference of Auxin Transport Mediated by *PIN1* Gene Family in Soybean and Cowpea

**FIGURE 1:**
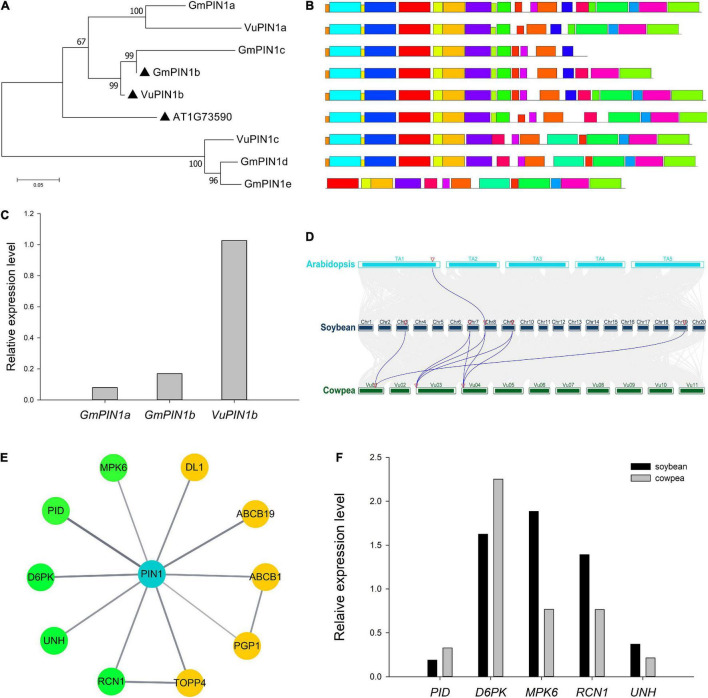
Analysis of PIN1 and its interaction proteins in soybean and cowpea. **(A)** Phylogenetics tree of *PIN1* gene family. **(B)** Motif analysis of PIN1 proteins. **(C)** Relative expression levels of targeted *PIN1* in soybean and cowpea flowers. **(D)** Collinearity analysis of *PIN1* gene family. **(E)** The network of proteins interacted with PIN1 in *Arabidopsis* predicted from the STRING database. The proteins with similar expression levels in soybean and cowpea were marked by yellow color, while those with significantly different expression levels in the two crops were marked by green color. **(F)** Comparison of the relative expression levels of genes, interacted with *PIN1*, between soybean and cowpea. As described by Zhang et al. (2018), the relative expression levels was calculated.

Among all the above OGs, five *GmPIN1* genes in soybean and three *VuPIN1* genes in cowpea were identified. The genes *GmPIN1a*, *GmPIN1b*, *GmPIN1c*, *VuPIN1a*, *VuPIN1b*, and *A. thaliana PIN1* were found to be in one gene family, and *GmPIN1b* and *VuPIN1b* had the highest relative expression levels in the flowers of soybean and cowpea (Table 1) and the closest genetic distance to *A. thaliana PIN1* gene (Figure 1A). All the *PIN1* genes were predicted by the Wolf PSORT software to be localized in the plasma membrane.

**TABLE 1:** Homologous genes of *PIN1* gene family in soybean, cowpea, and *Arabidopsis thaliana*.

Subfamily	Gene family in soybean			Gene family in cowpea			Gene name in *Arabidopsis*
	Gene ID	Gene name	Relative expression level	Gene ID	Gene name	Relative expression level	
1	*Glyma.08G054700*	*GmPIN1a*	0.079	*Vigun03g003500*	*VuPIN1a*	NA	*PIN1*
	*Glyma.07G102500*	*GmPIN1b*	0.169	*Vigun04g031500*	*VuPIN1b*	1.026	
	*Glyma.09G176300*	*GmPIN1c*	0.137				
2	*Glyma.03G126000*	*GmPIN1d*	NA	*Vigun01g105100*	*VuPIN1c*	0.065	−
3	*Glyma.19G128800*	*GmPIN1e*	NA	−	−	−	−

In the conserved motif analysis via the MEME software, almost all the PIN1 proteins were structurally relatively conserved (Figure 1B), except for GmPIN1c and GmPIN1e, which may be the repeat proteins with the lack of the sequences at the 3′ end of GmPIN1b and at the 5′ end of GmPIN1d, respectively. In the comparison of relative expression levels, *GmPIN1a* and *GmPIN1b* had significantly lower relative expression levels than *VuPIN1b* (Figure 1C). In the collinearity analysis in *Arabidopsis*, soybean, and cowpea via the TBtools software (Chen et al., 2020), *GmPIN1a* was collinear with *A. thaliana PIN1* gene; *GmPIN1d* and *GmPIN1e* were collinear with *VuPIN1c*; and *GmPIN1a*, *GmPIN1b*, and *GmPIN1c* were collinear with *VuPIN1a* and *VuPIN1b*. The results were consistent with those in the evolutionary tree (Figure 1D).

### Comparison of Proteins Interacted With *PIN1*, *CKX3*, and *CKX5* Between Soybean and Cowpea

Ten proteins in *A. thaliana* were found in STRING database to be interacted with *A. thaliana PIN1* based on experimental evidence. These proteins were used to mine homologous genes in soybean and cowpea. If one protein had multiple homologous genes in soybean or cowpea, the gene with the highest relative expression level was selected. In the large gene family of *TOPP4*; meanwhile, further phylogenetic analysis was conducted to determine the closest homologous relationship (Supplementary Figure 1A). The relative expression levels of homologous genes selected in soybean and cowpea were compared to find the differences of these homologous genes in soybean and cowpea. As a result, *MPK6*, *PID*, *D6PK*, *UNH*, and *RCN1* had significant differences of relative expression levels in soybean and cowpea (Figure 1F), while *ABCB1*, *ABCB19*, *DL1*, *PGP1*, and *TOPP4* had similar relative expression levels in soybean and cowpea (Figure 1E and Supplementary Table 1). Here we focused on the first five proteins.

First, we discussed their functions. *PID* and *D6PK* have been confirmed to positively regulate auxin transport through phosphorylation at the PIN1 serine S1 (S231), S2 (S252), S4 (S271), and S3 (S290), which plays an important role in ovular formation (Benjamins et al., 2001; Lee and Cho, 2006; Zourelidou et al., 2014). The S1 to S4 phosphate sites in *A. thaliana* were also found in the five soybean and cowpea PIN1 proteins, which are in the same OG as *A. thaliana* PIN1 (Supplementary Figure 2). *RCN1* is found to inhibit the phosphorylation of PIN1 protein to antagonize the polar transport of auxin (Rashotte et al., 2001; Zhou et al., 2004; Michniewicz et al., 2007; Dai et al., 2012), while MPK6-mediated phosphorylation of PIN1 leads to the loss of the plasma membrane localization of PIN1, affecting auxin polar transport (Dory et al., 2018). Up to now, only one phosphate site of *MPK6*, S337, has been confirmed (Jia et al., 2016), and this site was found to be conserved in soybean and cowpea PIN1 proteins (Supplementary Figure 2). *UNH* is important in reducing PIN1 expression level in marginal cells, possibly through the localization of PIN1 into vacuoles for degradation (Pahari et al., 2014).

Then, we compared their relative expression levels. As a result, *PID* and *D6PK*, which are conducive to the phosphorylation of PIN1, had lower relative expression levels in soybean than in cowpea, while *RCN1* and *UNH*, which affect plasma membrane localization, and *MPK6*, which affects the phosphorylation of PIN1, had higher relative expression levels in soybean than in cowpea. We speculated that an important reason for the SNPP difference between soybean and cowpea lies in the difference of relative expression level of *PIN1*. Owing to the difference of relative expression levels of genes encoding the interaction proteins of PIN1, PIN1 protein in soybean was less located on plasma membrane, and there was lower phosphorylation level in soybean. Thus, lower auxin transport efficiency results in less auxin maximum zone and fewer ovules and SNPP.

**FIGURE 2:**
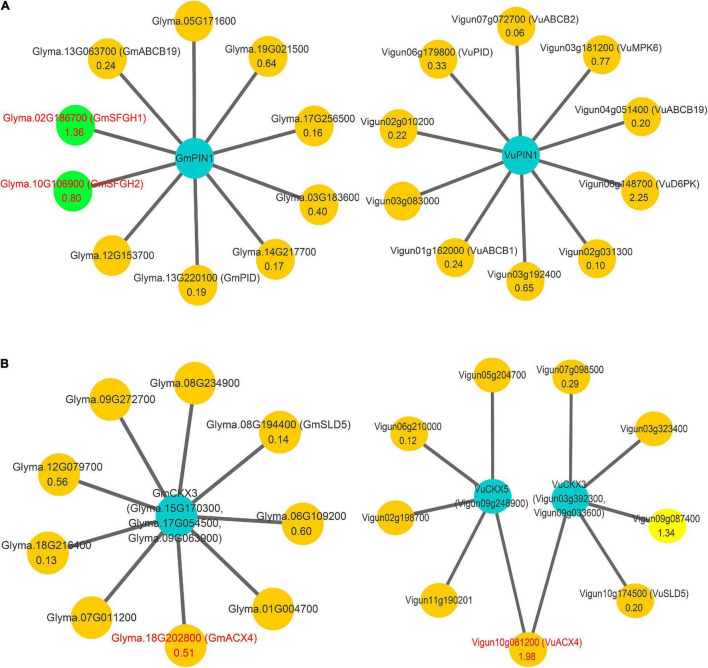
The interaction networks on *PIN1*, *CKX3*, and *CKX5* in soybean and cowpea. **(A)** The interaction networks on *GmPIN1* and *VuPIN1*. **(B)** The interaction networks on *GmCKX3*, *VuCKX3*, and *VuCKX5*. All the genes that encode serine hydrolase in soybean and short-chain acyl-CoA oxidase in cowpea were marked by red color and green background.

More importantly, the above method was used to predict the proteins that interact with *PIN1*, *CKX3*, and *CKX5* homologies in soybean and cowpea. The results are shown in Figure 2A and Supplementary Table 7. In the proteins interacted with *PIN1* homologies, two soybean proteins, *GmABCB19* (*Glyma.13G063700*) and *GmPID* (*Glyma.13G220100*), were consistent with those in *Arabidopsis*, while six cowpea proteins, *VuABCB1 (Vigun01g162000)*, *VuABCB2 (Vigun07g072700)*, *VuABCB19 (Vigun04g051400)*, *VuMPK6 (Vigun03g181200)*, *VuD6PK (Vigun06g148700)*, and *VuPID (Vigun06g179800)*, were consistent with those in *Arabidopsis*. Based on the functions and annotations of these interacted genes, they commonly focus on auxin transport and serine phosphorylation. Interestingly, *Glyma.02G186700* and *Glyma.10G106900* with high expression levels in soybean ovary may be involved in the hydrolysis of serine, which is the active site of *PIN1*, because their homology *AT2G41530* (AtSFGH) had been proven to encode a serine hydrolase in *Arabidopsis* (Cummins et al., 2006). *CKX3* and *CKX5* homologies in the two legumes commonly focus on embryo development and substance metabolism (Figure 2B). *GmSLD5 (Glyma.08g194400*, and *Glyma.07g011200)* and *VuSLD5 (Vigun10g174500)* are the homologies of *AT5G49010 (SLD5)*, whose mutant can cause defective embryo development in *Arabidopsis* (Meinke, 2020). Another pair of genes, *GmACX4 (Glyma.18g202800)* and *VuACX4 (Vigun10g081200)*, are the homologies of *ACX4 (AT3G51840)*, which encodes a short-chain acyl-CoA oxidase. *ACX4* is essential at early embryo development stages, and the *acx3acx4* double mutants abort during the first embryo development phase (Rylott et al., 2003). *VuACX4* (1.98) had much higher relative expression level than *GmACX4* (0.51). This may lead to better embryo development and more seeds in cowpea than in soybean. Thus, we infer that the hindrance of ovular formation via serine hydrolysis in soybean and the promotion of embryo development via *VuACX4* in cowpea may be responsible for the SNPP difference in the two legumes.

### Difference of Cytokinin Degradation Mediated by *CKX* Gene Family Between Soybean and Cowpea

#### Phylogenetic and Structural Analyses of *CKX* Gene Family

**FIGURE 3:**
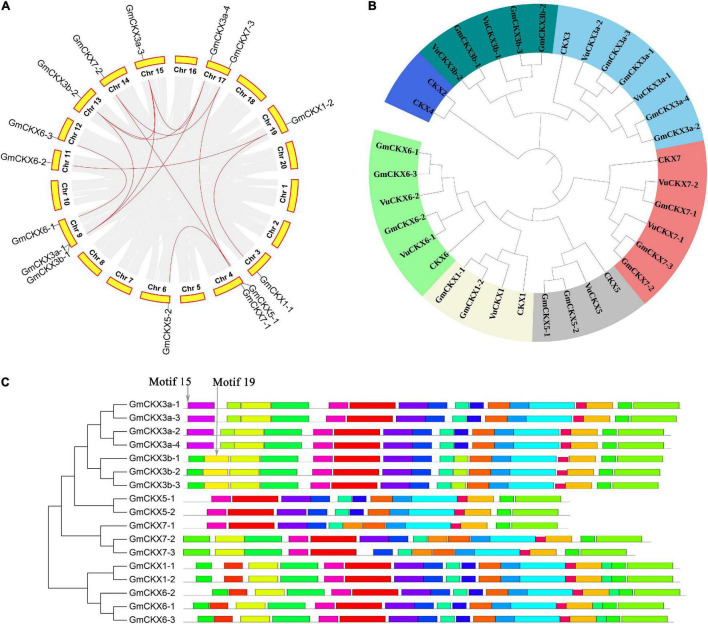
Bioinformatics analysis of *CKX* gene family in soybean. **(A)** Collinearity analysis of *CKX* gene family in soybean. **(B)** Phylogenetics tree of *CKX* gene family in *Arabidopsis*, *Glycine max*, and *Vigna unguiculata*. **(C)** The conserved motifs of *GmCKX* gene family.

Based on the protein sequence homology, the collinearity of gene sequences, and seven *A. thaliana CKX* genes, it was determined that a total of 17 soybean *CKX* genes and 10 cowpea *CKX* genes were in the same *CKX* gene family (Figures 3A,B). In the gene family, two kinds of soybean genes were homologous to the *CKX3 Arabidopsis* gene, higher homologous genes were named *CKX3a*, and other ones generated by tandem duplication were named *CKX3b* (Table 2). This tandem duplication was also found in acacia bean, *Medicago truncatula* (*Medtr4G126160*) and chickpea rather than in oilseed rape and rice. We deduced that the tandem duplications of *CKX3* are a specific event experienced in the evolutionary process of leguminosae.

**TABLE 2:** Homologous genes of *CKX* gene family in soybean, cowpea, and *Arabidopsis thaliana*.

*Subfamily*	*Gene family in Arabidopsis*		*Gene family in soybean*		*Gene family in cowpea*	
	Gene ID	Gene name	Gene ID	Gene name	Gene ID	Gene name
*1*	*AT2G41510*	*CXK1*	*Glyma.03G133300*	*GmCKX1-1*	*Vigun01g111600*	*VuCKX1*
			*Glyma.19G135100*	*GmCKX1-2*		
*2*	*AT2G19500*	*CKX2*				
	*AT4G29740*	*CKX4*				
*3*	*AT5G56970*	*CKX3*	*Glyma.09G063900*	*GmCKX3a-1*	*Vigun03g392300*	*VuCKX3a-1*
			*Glyma.13G104700*	*GmCKX3a-2*	*Vigun09g033600*	*VuCKX3a-2*
			*Glyma.15G170300*	*GmCKX3a-3*		
			*Glyma.17G054500*	*GmCKX3a-4*		
*4*	*AT1G75450*	*CKX5*	*Glyma.04G028900*	*GmCKX5-1*	*Vigun09g248900*	*VuCKX5*
			*Glyma.06G028900*	*GmCKX5-2*		
*5*	*AT3G63440*	*CKX6*	*Glyma.09G225400*	*GmCKX6-1*	*Vigun01g036800*	*VuCKX6-1*
			*Glyma.11G149100*	*GmCKX6-2*	*Vigun11g212700*	*VuCKX6-2*
			*Glyma.12G011400*	*GmCKX6-3*		
*6*	*AT5G21482*	*CKX7*	*Glyma.04G055600*	*GmCKX7-1*	*Vigun08g041000*	*VuCKX7-1*
			*Glyma.14G099000*	*GmCKX7-2*	*Vigun09g220900*	*VuCKX7-2*
			*Glyma.17G225700*	*GmCKX7-3*		
*7*			*Glyma.09G063500*	*GmCKX3b-1*	*Vigun03g392200*	*VuCKX3b-1*
			*Glyma.13G104600*	*GmCKX3b-2*	*Vigun09g033500*	*VuCKX3b-2*
			*Glyma.17G054600*	*GmCKX3b-3*		

In the domain analysis of the CKX proteins, these proteins contained cytokinin-bind and FAD binding 4 domains, which affect the binding of CKX proteins to cytokinins and FAD cofactors, respectively. Note that the lack of the first half of FAD binding domain sequences in GmCKX5-1, GmCKX5-2, and GmCKX7-1 may affect the binding of these proteins with FAD cofactors, and thus affect their functions and expression levels (Supplementary Figure 3).

In the phylogenetics tree, all the above 34 *CKX* genes were divided into seven classes, including *CKX1*, *CKX2* and *CKX4*, *CKX3a*, *CKX5*, *CKX6*, *CKX7*, and *CKX3b* classes (Figure 3B). Among these classes, *CKX3b* is close to *CKX3a*, and *CKX1* is close to *CKX6* in evolutionary relationship (Figure 3B). For the structural analysis of 17 *GmCKX* genes, all the structural information is shown in Supplementary Figure 4. We found that *GmCKX1* and *GmCKX3a* gene families had the highest similarity in the number, distribution, and length of CDS. In the motif analysis of the 17 *CKX* genes in soybean using MEME online tool, 23 conserved motifs were identified. Among these motifs, GmCKX6 and GmCKX1 had the same motif due to having similar function, GmCKX5 had no specific motifs, and the other GmCKX families had some specific motifs, i.e., motif15 is specific to GmCKX3a proteins, and motif19 is specific to GmCKX3b proteins. These specific motifs may be related to their specific functions (Figure 3C).

#### Expression Pattern and Expression Level Analyses of *CKX* Gene Family

**FIGURE 4:**
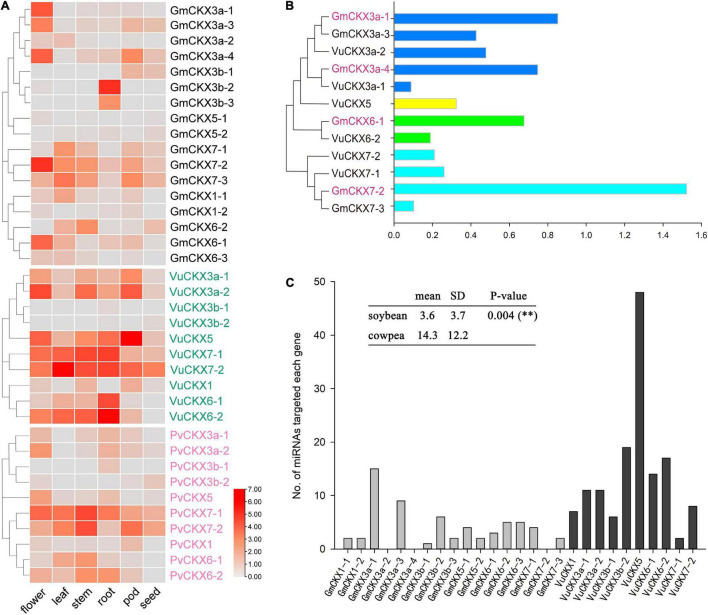
Expression patterns and regulation of *CKX* gene family. **(A)** Expression levels of *CKX* gene family in flower, leaf, stem, root, pod, and seed of soybean, cowpea and kidney bean. **(B)** Relative expression levels of *CKX* gene family in soybean and cowpea flowers. **(C)** The numbers of miRNAs targeted each *CKX* gene.

We downloaded and analyzed the expression levels of these *CKX* genes in different tissues of soybean and cowpea. In soybean, *GmCKX5* with incomplete domain and *GmCKX3b-1* were hardly expressed in each tissue, while *GmCKX3a*, *GmCKX7-2*, and *GmCKX6-1* were expressed mainly in flowers, and *GmCKX3b-2* and *GmCKX3b-3* were expressed in roots. Meanwhile, *VuCKX5* with a complete domain in cowpea was expressed in flowers, roots, and pods, *VuCKX6* and *VuCKX7* genes were highly expressed in roots, *VuCKX3a* gene was expressed in flowers, and *VuCKX3b* gene was not expressed in any tissues (Figure 4A). In addition, we identified 10 *CKX* genes in kidney bean, which correspond to 10 *VuCKX* genes in the evolutionary tree (Supplementary Figure 1B and Supplementary Table 4). Their expression patterns in various tissues were the same as those in cowpea. For example, *PvCKX3b* were hardly expressed in all tissues, *PvCKX7-1* and *PvCKX6-2* were highly expressed in roots, and PvCKX3a was expressed in flowers (Figure 4A).

Recently, Wang et al. (2021) identified the function of *Medtr4G126160* in *Medicago truncatula*, which is the highest homology with *GmCKX3b-2* and *GmCKX3b-3* (Supplementary Table 3). In detail, the *Medtr4G126160* mutant significantly reduced main root length and increased the number of lateral roots, indicating the important role of *Medtr4G126160* in root development. In this study, *CKX3b* genes were found to have two expression patterns in different *leguminosae* crops. In soybean and *Medicago truncatula*, the *CKX3b* genes were highly expressed in roots, which may play an important role in root development, while, in cowpea and kidney bean, the *CKX3b* genes were hardly expressed in various tissues, indicating the functional differentiation of *CKX3b* tandem duplication in plant evolution.

In addition, we compared the relative expression levels of the *CKX* gene family in soybean and cowpea flowers (Figure 4B). As a result, although *GmCKX5* with incomplete structure domains had significantly lower relative expression level in soybean than in cowpea, *CKX3*, *CKX6*, and *CKX7* had much higher relative expression levels in soybean than in cowpea. These highly expressed *GmCKX* genes, especially the *GmCKX3a* genes, reduce cytokinins content in soybean in the process of ovule formation. Less cytokinins result in low expression of auxin efflux carrier *PIN1*. Thus, there are lower number of ovules and SNPP (Bartrina et al., 2011; Bencivenga et al., 2012; Zuñiga-Mayo et al., 2018).

#### Prediction of miRNA and *ROCK1* Gene Regulation in *CKX* Gene Family

To predict all the miRNAs for targeted soybean and cowpea *CKX* genes via the online software PsRNATarget, we downloaded 756 soybean miRNA sequences from the miRBase database and 656 cowpea miRNA sequences from Martins et al. (2020). As a result, 62 miRNAs were predicted to be targeted with soybean *CKX* genes, while 144 miRNAs were predicted to be targeted with cowpea *CKX* genes (Figure 4C). In the cowpea *CKX* gene family, *VuCKX5* was predicted to be regulated by 48 miRNAs (maximum), while *VuCKX7-1* was predicted to be regulated by 2 miRNAs (minimum). In the soybean *CKX* gene family, *GmCKX3-1* was predicted to be regulated by 15 miRNAs (maximum), while no miRNAs were predicted to regulate *GmCKX3a-2*, *GmCKX3a-4*, and *GmCKX7-2* (Figure 4C). We speculated that less miRNA regulation in soybean may be one reason for relatively high expression levels of soybean *CKX* genes.

In Niemann et al. (2015), ROCK1 was a positive regulator of CKX protein activity in *Arabidopsis thaliana*, while in this study, soybean *ROCK1* genes (*GmROCK1* and *Glyma.08G135800*) had much higher relative expression level than cowpea *ROCK1* genes (*VuROCK1* and *Vigun03G076200*). This may result in higher activity of soybean CKX protein than cowpea CKX protein, which would enhance soybean cytokinin degradation.

### Expansion Patterns of the *CKX* Gene Family in Soybean

Using the database PlantDGD (Qiao et al., 2019), we obtained 13 pairs of duplicate genes in soybean, which were consistent with our collinearity analysis results. Among these duplicate genes, nine pairs were normal and four pairs were abnormal, namely, three pairs of *GmCKX3a* and *GmCKX3b* on chromosomes 9, 13, and 17 owing to the mismatch in collinearity analysis, and one pair of *GmCKX6-2* and *GmCKX1-2* (Supplementary Table 6). Here, we replaced *GmCKX3b* with its adjacent *GmCKX3a* and calculated the Ks values of these gene pairs to estimate their replication times (Supplementary Table 5). As a result, the duplications for six, six, and one pairs of *GmCKX* genes occurred, respectively, between 8 and 18 mya, between 70 and 74 mya, and between 133 and 169 mya. This indicates that most *GmCKX* duplications occurred at 10–15 mya [a soybean-specific whole genome duplication (WGD) event] and 59 mya (a legume-specific WGD event), and individual *GmCKX* duplications occurred at approximately 150 mya.

Using the database PlantDGD (Qiao et al., 2019), we checked the fragment duplications of *CKX* gene family in kidney bean and *Arabidopsis*. As a result, a pair of *PvCKX1* and *PvCKX6* was obtained, while three pairs of possible fragment replicators *CKX2* and *CKX3*, *CKX2* and *CKX4*, and *CKX3* and *CKX4* in *Arabidopsis* were observed. Thus, we speculated that *CKX2* and *CKX4* were a segmental duplication event that occurred in other species after legume differentiation, while one copy of *CKX6* gene was a segmental duplication of *CKX1* in legumes. The *CKX6* copy from *CKX1* is different from the other *CKX6* copies in expression pattern. The former was not expressed in flowers, while the latter was expressed in flowers.

### Comparison of Relative Expression Levels for *PIN1* and *CKX* Gene Families in Soybean and Kidney Bean

To confirm whether the above SNPP relationship and the difference of expression levels of *PIN1* and *CKX* gene families exists in kidney bean, we compared their relative expression levels in soybean and kidney bean. As a result, *Phvul.004G150600* had much higher relative expression level in kidney bean flowers than *GmPIN1a* and *GmPIN1b* in soybean flowers, while *GmCKX* gene family had higher relative expression levels in flowers than *PvCKX* gene family (Supplementary Figure 5). The results were consistent with those in soybean and cowpea. The differences of relative expression levels of *PIN1* and *CKX* gene families in soybean and cowpea, along with the differences in soybean and kidney bean, may be an important reason for the SNPP difference.

### Comparison of Seed Number per Pod-Related Interaction Networks in Soybean and Cowpea

**FIGURE 5:**
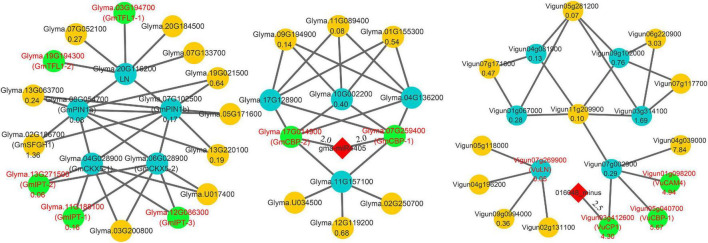
Main interaction networks on SNPP-related genes in soybean and cowpea. All the genes that encode Isopentenyltransferase, TERMINAL FLOWER 1, and calmodulin and calcium-binding proteins were marked by red color characters and green background.

Candidate SNPP genes in this and previous (Schwarz et al., 2020; Fang et al., 2021) studies and known gene *Ln* were used to construct interaction networks in soybean and cowpea. All the results are shown in Figure 5 and Supplementary Table 7. In soybean network, low SNPP may be due to two reasons. First, four-seed-pod-related gene *Glyma.10G002200*, in Fang et al. (2021), was interacted with *GmCBP-1* and *GmCBP-2*, while its homology *Vigun07g002900* in cowpea was interacted with *VuCBP-1*, *VuCP1*, and *VuCAM4.* The five interacted genes in soybean and cowpea belong to the calmodulin and calcium-binding protein gene (*CBP)* family. In previous studies, calmodulin and calcium-binding proteins in plants not only directly affected SNPP (Midhat et al., 2018), but were also critical for the biosynthesis of brassinosteroid (Du and Poovaiah, 2005), which plays an important role in determining the number of ovules and seeds via positive regulator *BZR1* (Huang et al., 2013). However, *VuCBP-1* (5.67), *VuCP1* (4.30), and *VuCAM4* (4.94) had much higher relative expression levels than *GmCBP-1* (0.38) and *GmCBP-2* (0.90), which are inhibited by gma-miR4405. Although *Vigun03g412600* was inhibited by 016048_minus, its binding degree was relatively low (Supplementary Table 8). Thus, we speculated that the high expression may increase SNPP in cowpea.

Then, SNPP-related genes, *GmCKX5-1* and *GmCKX5-2*, were interacted with soybean genes *GmIPT-1* (*Glyma.11G188100*), *GmIPT-2* (*Glyma.13G271500*), and *GmIPT-3* (*Glyma.12G086300*). Their homologies in *Arabidopsis* all belong to Isopentenyltransferase (*IPT*) gene family, which plays an important role in cytokinin biosynthesis (Miyawaki et al., 2004, 2006), and overexpressing *IPT* can increase the levels of endogenous cytokinins (Décima Oneto et al., 2016). Their low expression levels in soybean may lead to a decrease in cytokinin synthesis, which further affects ovule numbers in soybean. The negative regulation was not found in cowpea.

In addition, a known SNPP gene *Ln* was interacted with *Glyma.03G194700* (*GmTFL1-1*) and *Glyma.19G194300* (*GmTFL1-2*), for which their *Arabidopsis* homology *AT5G03840* (*TFL1*) determines seed size, and loss-of-function mutants exhibit a large seed phenotype (Zhang et al., 2020). Low expression levels of *GmTFL1-1* (0.10) and *GmTFL1-2* (0.01) may lead to large seed in soybean. The interacted network was not found in cowpea.

### Comparison of Yield-Related Gene Network in Soybean and Cowpea

**FIGURE 6:**
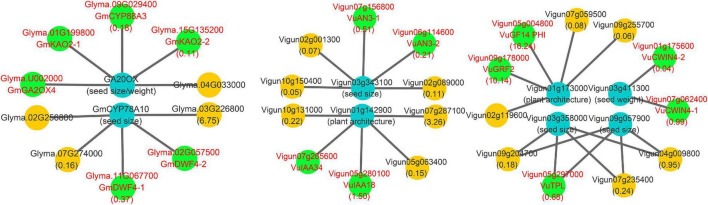
Main interaction networks on yield-related genes in soybean and cowpea. All the genes that encode *CYP88A* gene family, *gibberellin oxidase*, *22*α *hydroxylase*, *14-3-3* gene family, *CWIN4*, and *AN3* were marked by red color characters and green background.

In this study, all the known yield-related soybean genes in Zhang et al. (2021), along with the above known and candidate SNPP genes, were used to construct a comprehensive network for the two legumes. The results are shown in Figure 6 and Supplementary Table 9.

In the network, some negative regulation was found in soybean. First, known seed size/weight gene *Glyma.07G081700* was found to interact with *Glyma.09G029400*, *Glyma.01G199800*, and *Glyma.15G135200* of cytochrome P450 subfamily 88A (*CYP88A*) gene family, which catalyzes the conversion of KA (ent-kaurenoic acid) to GA12 (the precursor of all gibberellins) and catalyzes three steps of gibberellin biosynthesis pathway in *Arabidopsis* (Helliwell et al., 2001; Regnault et al., 2014). It should be noted that GA was found to negatively regulate the number of ovules in Fuentes et al. (2012) and Carrera et al. (2012). Then, *Glyma.07G081700* was found to interact with *Glyma.U002000* that encodes gibberellin oxidase, whose expression decreases cytokinin activity (Jasinski et al., 2005). Finally, known seed-size-gene *Glyma.05g019200* was found to interact with *Glyma.11G067700* and *Glyma.02G057500*, which encode 22α hydroxylase that is an inhibitor of Brassinosteroid (BR) biosynthesis in *Arabidopsis* (Fujiyama et al., 2019). The negative regulation may result in lower SNPP in soybean than in cowpea.

Meanwhile, some positive regulation was found in cowpea. First, the homology *Vigun01g142900* of known plant-architecture gene *Glyma.19G164600* was found to be interacted with *Vigun07g265600* and *Vigun05g280100*, while the homology *Vigun03g358000* of known seed size gene *Glyma.17G112800* was found to be interacted with *Vigun05g297000*. *Vigun07g265600* (*VuIAA34*), *Vigun05g297000* (*VuTPL*), and *Vigun05g280100* (*VuIAA18*) focused on auxin synthesis and transport (Liscum and Reed, 2002). Then, the homology *Vigun01g173000* of known plant-architecture gene *Glyma.19G194300* was found to be interacted with *Vigun05g004800* and *Vigun09g178000* in 14-3-3 gene family, which is involved in *PIN* auxin carrier, auxin transport-related development, and brassinosteroid signal transduction (Gampala et al., 2007; Keicher et al., 2017). In this study, *Vigun05g004800* and *Vigun09g178000* were found to have high expression levels at ovule developmental stages. Third, the homology *Vigun03g411300* of known seed-weight gene *Glyma.17g036300* was found to be interacted with cell wall invertase genes *Vigun01g175600* and *Vigun07g062400*, in which their homology *AT2G36190* (*CWIN4*) may regulate ovule formation by modulating downstream auxin signaling and MADS-box transcription factors in *Arabidopsis* (Liao et al., 2020). Finally, the homology *Vigun06g114600* of known seed size gene *Glyma.17G112800* was found to be interacted with *Vigun06g114600* and *Vigun07g156800*, in which their homology *AT5G28640* (*AN3*) can regulate seed embryo development together with *AT1G55600* (*MINI3*), and its mutant line had lower seed/silique number, silique length, and seed/silique weight than wild-type plants (Meng et al., 2016). The positive regulation results in higher SNPP in cowpea than in soybean.

Known seed size or weight genes *Glyma.01G061100* (*GmCYP78A70*), *Glyma.02G119600* (*GmCYP78A57*), *Glyma.19G240800* (*GmCYP78A72*), and *Glyma.05G019200* (*GmCYP78A10*) belong to the *CYP78A* gene family, which is found to be associated with seed size in *Arabidopsis* (Adamski et al., 2009; Fang et al., 2012). In cowpea, there were only two homologous copies *Vigun02g047800* and *Vigun03g343100* of the four soybean genes. We found that the first three soybean genes had high expression levels at middle and later seed development stages, while all the two cowpea copies had low expression levels (Supplementary Table 10). This may explain why soybean seed is larger than cowpea seed.

In summary, species-specific traits in crops may be derived from species-specific gene networks.

## Discussion

### Molecular Mechanisms for Seed Number per Pod Difference in Soybean and Cowpea

**FIGURE 7:**
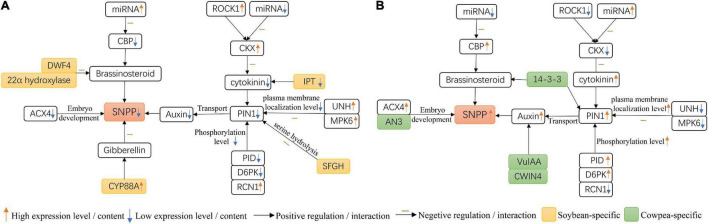
Molecular mechanisms for the difference of seed number per pod (SNPP) in **(A)** soybean and **(B)** cowpea.

In this study, we observed four interesting results. The results are showed in Figure 7. First, *PIN1* had lower expression level in soybean flowers than in cowpea flowers. Then, among the proteins, *PID*, *D6PK*, *RCN1*, *UNH*, and *MPK6* in *Arabidopsis*, that interacted with *PIN1* and their homologies were differentially expressed between soybean and cowpea, lower *PID* and *D6PK* and higher *RCN1* expression levels in soybean resulted in lower phosphorylation level in soybean flowers, while high *UNH* and *MPK6* expression levels decreased plasma membrane localization level in soybean flowers, as compared with those in cowpea flowers. Thus, we speculate that lower *PIN1* expression level and lower phosphorylation and plasma membrane localization levels derived from the above five differential expression proteins interacted with PIN1 make auxin transport efficiency lower in soybean flowers than in cowpea flowers, which forms a lower auxin maximum zone (Benková et al., 2003; Ceccato et al., 2013). Next, some differential genes in the interaction networks on *PIN1* and *CKX* gene families were found. *SFGH*, which hydrolyzes serine, was found in soybean rather than in cowpea, and *ACX4* had much higher relative expression level in cowpea than in soybean. These may lead to better embryo development and more seeds in cowpea. Finally, in the interaction networks on yield-related genes, higher expression levels of the *CBP* genes in cowpea, as compared with those in soybean, increase SNPP; low expression levels of three soybean-specific *IPT* genes may inhibit CK synthesis. The above results may lead to lower number of ovules and ultimately lower SNPP in soybean than in cowpea.

In a previous study, one locus for soybean four-seed pod was located by Yang et al. (2013) on chromosome 13. Around this locus, there was one candidate gene *GmMIF2* (*Glyma.13G063100*). Here we found that soybean gene *GmMIF2* had much higher relative expression level in flower than cowpea gene *VuMIF2* (*Vigun04g052300*). More importantly, their homologous gene *MIF2* (*AT3G28917*) in *Arabidopsis* was found to hinder its ovule development (Bollier et al., 2018). Thus, *MIF2* may be a potential gene for SNPP difference between soybean and cowpea.

In addition, we explained the reasons for low expression level of *PIN1* gene in soybean. In this study we observed another two interesting phenomena. First, *CKX* genes and their positive regulator *ROCK1* had higher relative expression levels in soybean flower than in cowpea flower. Then, fewer miRNAs were predicted to be targeted with soybean *CKX* genes than with cowpea *CKX* genes. These two results lead to higher cytokinin degradation level in soybean than in cowpea, which decreases cytokinin level in soybean (Supplementary Table 11) and affecting the expression level of *PIN1* in soybean (Bencivenga et al., 2012; Zuñiga-Mayo et al., 2018).

### Auxin Transport Efficiency Difference May Cause Seed Number per Pod Difference in Soybean and Cowpea

In previous studies, three aspects of low auxin transport efficiency in *Arabidopsis* have been observed. First, *PIN1* expression level affects auxin transport efficiency. Okada et al. (1991) compared the polar transport efficiency of auxin in the inflorescence tissue between *pin1* mutants and their wild type, indicating the significant decreases of polar transport efficiency between *pin1-1* (↓ 86%) and *pin1-2* (↓ 93%) mutants and their wild type. Then, plasma membrane localization level of *PIN1* affects auxin transport efficiency. Wisniewska et al. (2006) modified *PIN1* polarity and examined auxin translocation direction in *Arabidopsis thaliana*. As a result, *PIN1* polarity determines a primary direction in auxin transport of meristematic tissues. Finally, some kinases have been found to positively regulate auxin transport through *PIN1* phosphorylation in *Arabidopsis*, such as *PID* and *D6PK* (Benjamins et al., 2001; Lee and Cho, 2006; Zourelidou et al., 2014). In addition, *GmPIN1* is expressed and polarly localized in nodule primordium cells, and controls nodule formation by transporting auxin to form an auxin maximal zone in soybean (Gao et al., 2021). As we know, nodule primordium and ovule primordium are meristematic tissues. Thus, these findings provide evidence for the role of *GmPIN1* in the formation of soybean ovule primordium. In this study, we found that the relative expression level of *PIN1* gene was much lower in soybean flowers than in cowpea flowers, and the differences of relative expression levels of the above five proteins interacting with PIN1 might cause lower phosphorylation and plasma membrane localization levels in soybean than in cowpea. These results may cause lower auxin transport efficiency in soybean than in cowpea.

During ovule primordium formation, Benková et al. (2003) found that auxin accumulated in large amounts at the apex of ovule primordium, and this auxin maximum zone is a necessary condition for ovule formation. Bencivenga et al. (2012) showed that lower auxin transport efficiency significantly reduced ovule number per pistil from wild-type *Col-0* (48 ovules) to weak mutant *pin1-5* (9 ovules). Carlson and Lersten (2004) and Yang et al. (2017) observed that ovule number difference could cause SNPP difference. In summary, auxin transport efficiency difference in soybean and cowpea may cause lower formation efficiency of auxin maximum zone in soybean, resulting in lower ovule number and SNPP in soybean.

### *CKX* Genes Play an Important Role in Ovule Formation

Cytokinin dehydrogenase (CKX) can specifically degrade cytokinin, although cytokinin can increase ovules number by promoting *PIN1* expression (Bencivenga et al., 2012). Bartrina et al. (2011) compared single and double *CKX* gene mutations with their wild types in *Arabidopsis thaliana*. As a result, no significant change in the overall plant morphologies of single *CKX* gene mutants was observed, indicating the redundant role of *CKX* gene family. Multiple double mutations with *CKX3-1* allele could form more flowers, especially for *ckx3 ckx5* double mutant, which formed more ovules. This increased SNPP and led to 55% higher seed yield. Schwarz et al. (2020) obtained similar results in *Brassica napus*. In detail, compared with the wild type, the *bnckx3 bnckx5* sixfold mutant increased the number of flowers, ovule number per pistil, and pod numbers on main stem, increasing seed yield by 20–32%. These results suggest that *CKX* gene family plays an important role in ovule formation. In this study, we identified 17 soybean and 10 cowpea *CKX* genes. Among these genes, most were not expressed in flowers, and multiple copy genes *CKX3*, *CKX6*, and *CKX7* were much higher in soybean flowers than in cowpea flowers. These high expression *CKX* genes in soybean may lead to more cytokinin degradation and lower cytokinin content, decreasing *PIN1* expression level, ovule number per pistil, and SNPP.

At present, there have been limited studies on *CKX* gene regulator, and only one regulator was reported by Niemann et al. (2015). In detail, the enhanced CKX activity in 35S:CKX1, 35S:CKX2, and 35S:CKX3 plants was reduced through rock1 introgression by 90.5, 64, and 100%, respectively. Meanwhile, *rock1* mutant enhanced the activity of apical meristem and organ formation rate in *Arabidopsis thaliana*. Cytokinin content in inflorescence was increased and the numbers of flowers and pods on main stem were 50% higher in *rock1* mutant than in its wild type, which was very similar to the phenotypic changes in the *ckx3 ckx5* mutant. These results indicate that ROCK1 acts as a positive regulator of CKX protein. In this study, we found one *ROCK1* homologous gene in soybean or cowpea, and *ROCK1* had higher relative expression level in soybean flowers than in cowpea flowers. The higher *ROCK1* gene expression level may increase the activity of soybean CKX protein. In addition, fewer miRNAs were predicted to target *GmCKX* genes. In other words, less miRNA regulation may be an important reason for higher *GmCKX* expression level in soybean. Thus, higher *ROCK1* expression and fewer miRNAs enhanced cytokinin degradation by regulating *GmCKX*, so ovule number and SNPP in soybean decreased.

### Breeding by Design for Seed Number per Pod in Soybean

Molecular design breeding has been widely used in soybean with some success (Haun et al., 2014; Bao et al., 2019; Han et al., 2019; Le et al., 2020). As we know, cowpea has more SNPP and smaller seeds, and soybean has fewer SNPP and larger seeds. However, cowpea has significantly higher yield than soybean. We also notice that relatively few SNPP in wild and cultivated soybeans may hinder the increase of soybean yield. Therefore, it is possible and necessary to mine yield-related novel genes in cowpea to conduct molecular design breeding in soybean, especially in the current situation of very sharp contradiction between soybean supply and demand in China.

First, *Gmckx3a* quadruple mutant may be used to increase SNPP and yield in soybean. In previous studies, *ckx3 ckx5* mutant in *Arabidopsis* and *bnckx3 bnckx5* sixfold mutant in *Brassica napus* can increase their yields (Bartrina et al., 2011; Schwarz et al., 2020). In soybean, we found high expression of four *CKX3a* and no expression of two *CKX5* and three *CKX3* tandem-duplication-derived genes (*CKX3b*) in flowers (Figure 4). Thus, *ckx3a* quadruple mutant may be used to increase SNPP and soybean yield. In addition, *GmCKX6-1* and *GmCKX7-2* were found to be highly expressed in flowers in this study. Another possible way is to obtain *gmckx3a gmckx6* (or *gmckx7*) mutant.

Second, we can identify elite alleles of *GmCKX3a*, *GmCKX6* and *GmCKX7* from existing four-seed pod cultivars, and transfer these elite alleles into current excellent cultivars via cross and backcross approaches. This method has been confirmed to be effective in Zhu et al. (2020).

Third, we may interfere with the expression of *CYP88A* genes (*Glyma.09G029400*, *Glyma.01G199800*, and *Glyma.15G135200*) and increase DELLA protein to decrease GA content in soybean. Carrera et al. (2012) reported that inhibiting the synthesis and function of GA via DELLA protein in tomato can increase ovule number.

Finally, we may over-express the *CBP* genes in soybean (*Glyma.17G019400* and *Glyma.07G259400*) and transfer an excellent mutant gene *GmBZL2*(*P216L*) (GmBZL2*) into soybean (Zhang et al., 2016) to increase SNPP via enhancing the synthesis and signal transduction of BR.

## Data Availability Statement

The original contributions presented in the study are included in the article/Supplementary Material, further inquiries can be directed to the corresponding author/s.

## Author Contributions

Y-MZ conceived and supervised the study. L-ML, H-QZ, and KC carried out the experimental works and analyzed the data. L-ML, H-QZ, and Y-MZ wrote and revised the manuscript. All authors read and approved the final manuscript.

## Conflict of Interest

The authors declare that the research was conducted in the absence of any commercial or financial relationships that could be construed as a potential conflict of interest.

## Publisher’s Note

All claims expressed in this article are solely those of the authors and do not necessarily represent those of their affiliated organizations, or those of the publisher, the editors and the reviewers. Any product that may be evaluated in this article, or claim that may be made by its manufacturer, is not guaranteed or endorsed by the publisher.

## Footnotes

1
https://www.arabidopsis.org/index.jsp

2
http://www.soybase.org/

3
https://legumeinfo.org/data/public

4
https://phytozome.jgi.doe.Gov/phytomine/template.do?Name=Gene_Expression

5
https://www.ncbi.nlm.nih.gov/bioproject/?term=GSE42871

6
https://www.ncbi.nlm.nih.gov/bioproject/?term=prjna389300

7
http://www.mirbase.org/

8
http://www.megasoftware.net/

9
http://itol.embl.de/

10
http://gsds.gao-lab.org/index.php

11
https://pfam.xfam.org/search

12
https://wolfpsort.hgc.jp/

13
http://meme-suite.org/tools/meme

14
https://string-db.org/

15
http://plantgrn.noble.org/psRNATarget/analysis

16
http://pdgd.njau.edu.cn:8080/
