# Supplementary material for: Integrated Bioinformatics Analyses of PIN1, CKX, and Yield-Related Genes Reveals the Molecular Mechanisms for the Difference of Seed Number Per Pod Between Soybean and Cowpea
Source: Front Plant Sci. 2021 Nov 29;12:749902. doi: 10.3389/fpls.2021.749902 (PMC8667476; doi:10.3389/fpls.2021.749902)
Supplement: Supplementary file 1 [file Data_Sheet_1.PDF]

A

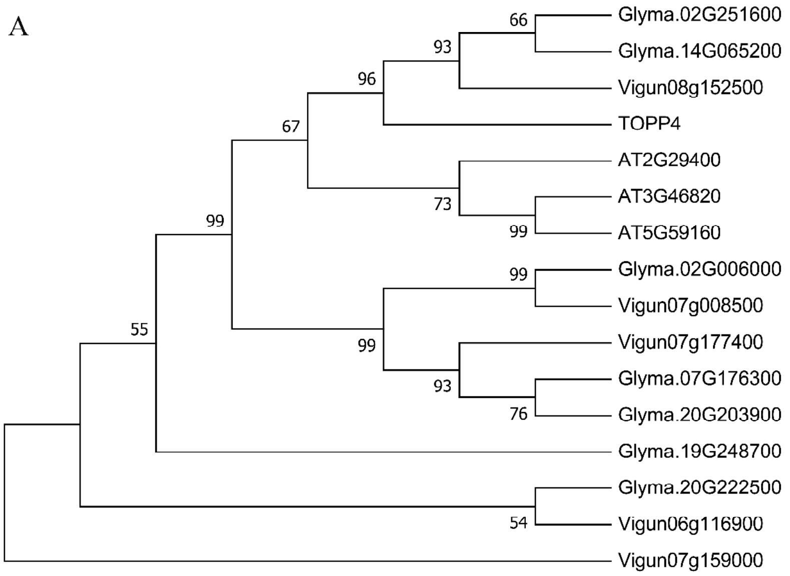

B

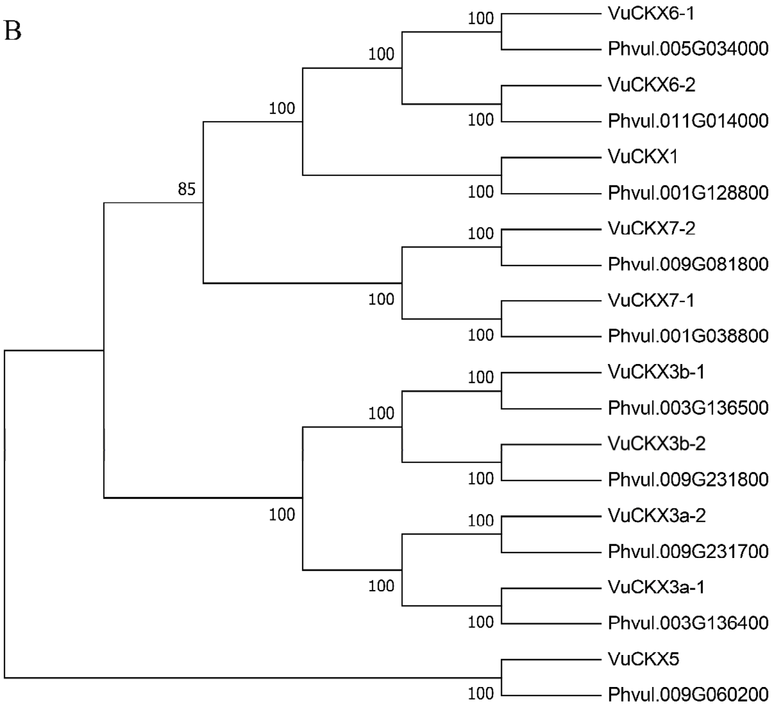

Supplementary Figure S1 | Phylogenetics trees of *TOPP4* gene family in *Arabidopsis*, soybean and cowpea (A) and *CKX* gene family in cowpea and kidney bean (B).

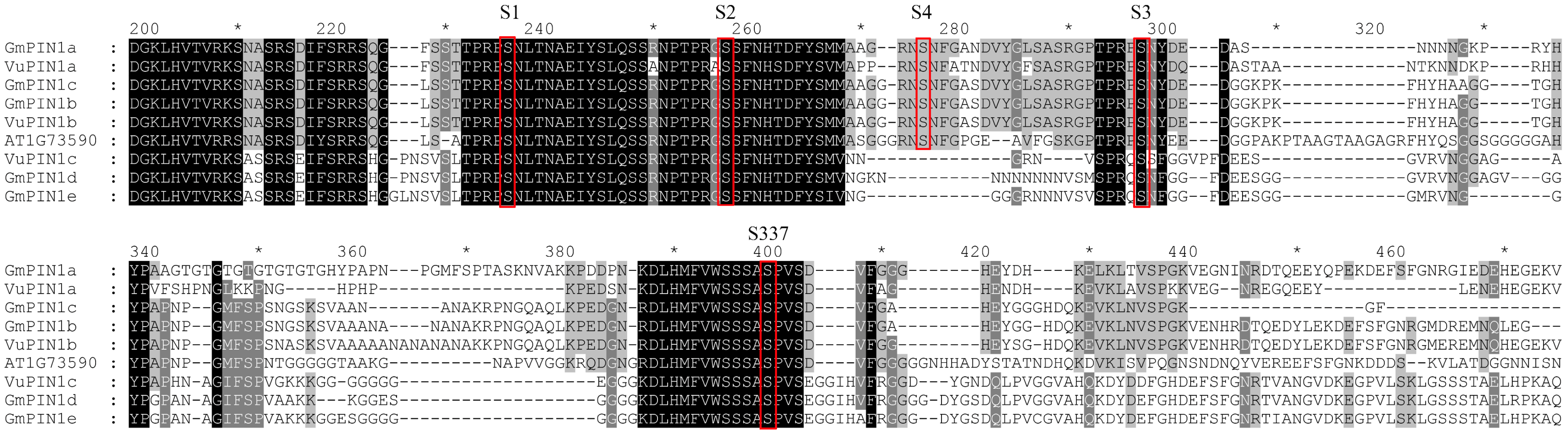

Supplementary Figure S2 │ Phosphoric acid sites of PID, D6PK, and MPK6 of PIN1 proteins in *Arabidopsis*, soybean and cowpea.

A The part of FAD binding 4 domain

```
                    *         80         *        100         *        120         *        140
VuCKX5    : PSEVVHPATAQDVARVVKAALESGFAVSARGHGHSINGQALIREKKGVVIEMGKG-----GSGVRVCEKGMYVDVWGGEL
GmCKX5-1  : ----------------------------------------MIKEKKGVVIEMGKSDSGEDGSSIRVSEKGMYVDVWGGKL
GmCKX5-2  : ------------------------MGKSDSGDNNDHNGDSNI---------------------IRVCEKGMYVDVWGGKL
```

B The domains of CKX5

VuCKX5:

GmCKX5-1 & GmCKX5-2:

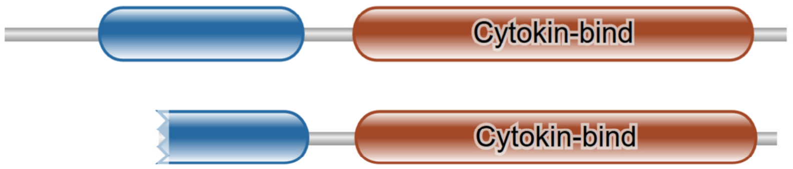

Supplementary Figure S3 | Protein sequences and structures of domains for VuCKX5, GmCKX5-1, and GmCKX5-2.

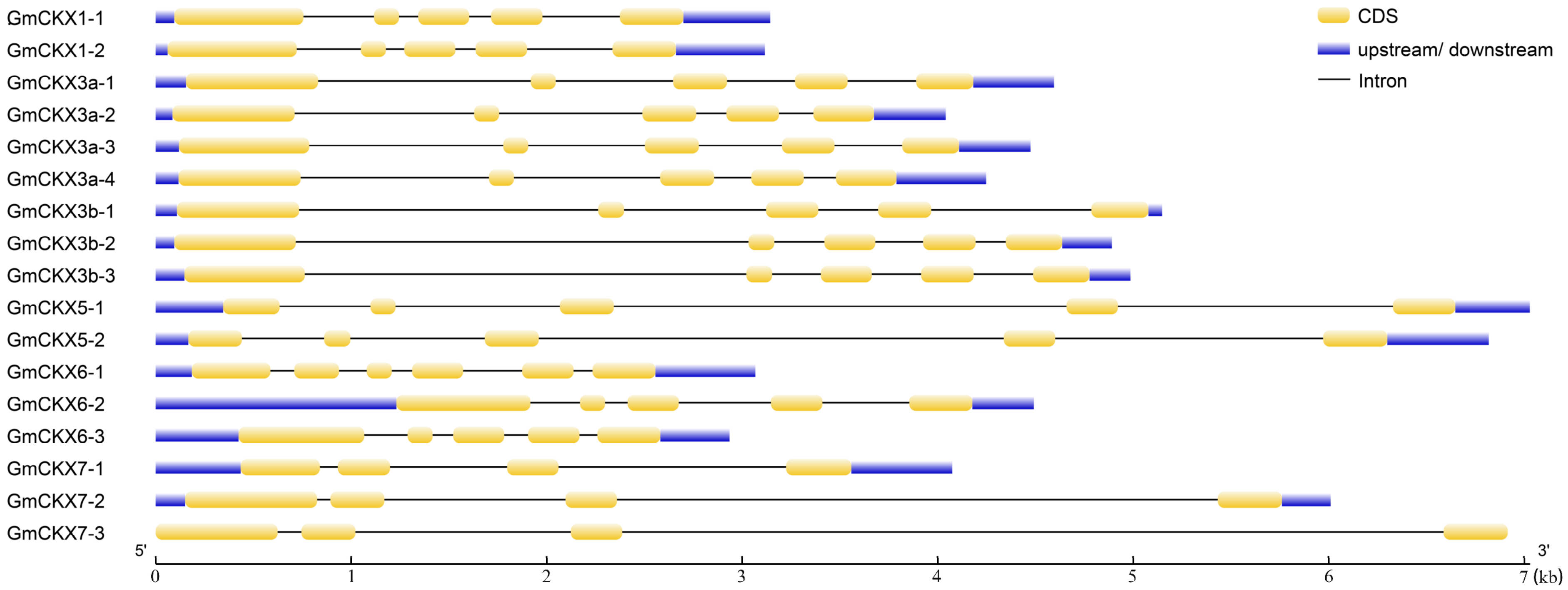

Supplementary Figure S4 | Exon-intron structures of *GmCKX* gene family.

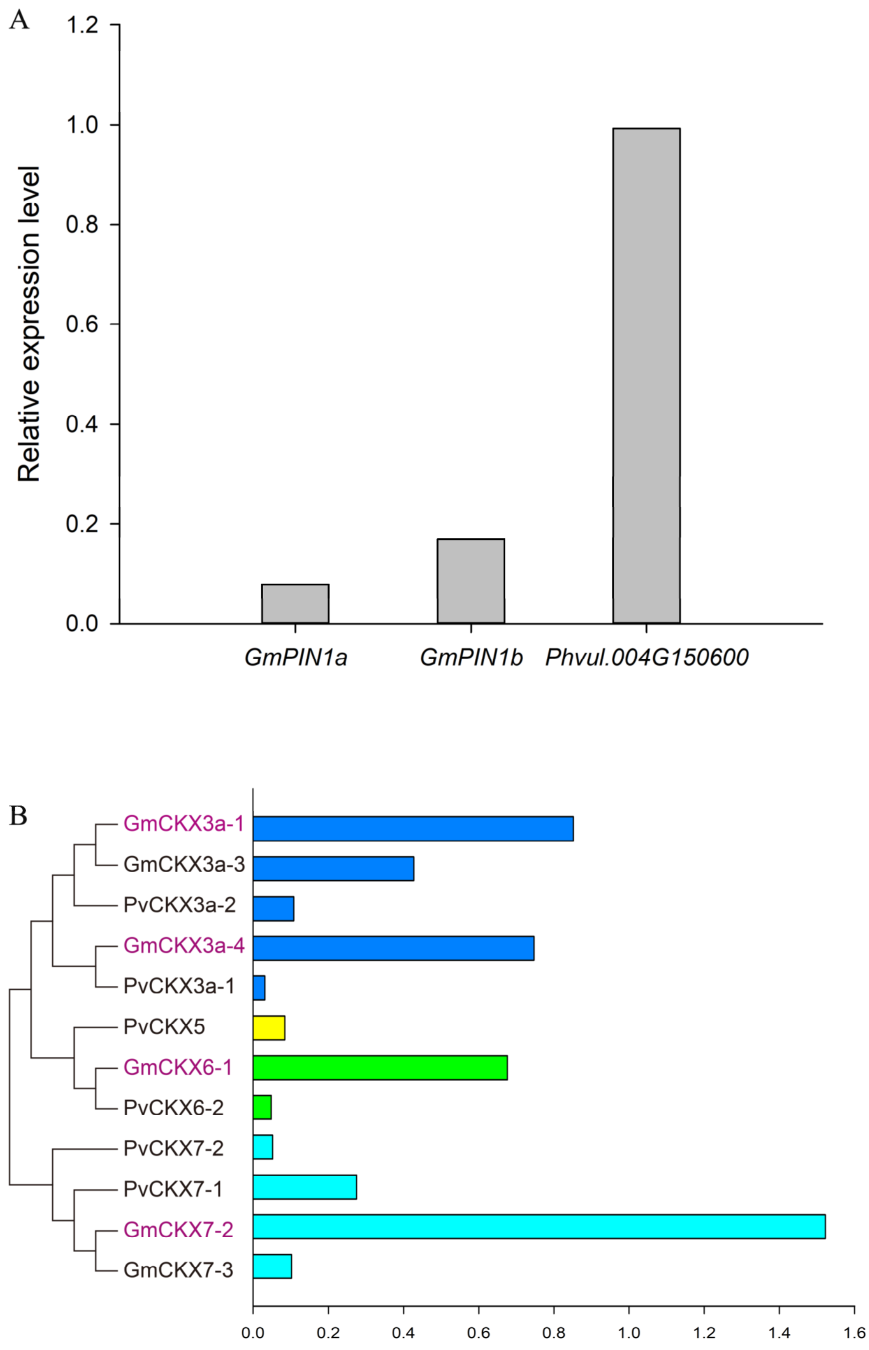

Supplementary Figure S5 │ Relative expression levels of targeted *PIN1* (A) and *CKX* gene families (B) in soybean and kidney bean flowers.

Supplementary Table S1 | Comparisons of the spread of counts of sequence similarity-based protein families across *Glycine max* (gma) and *Vigna unguiculata* (vun)

	**Unique for gma**	**Unique for vun**	**One copy for gma & multiple copies for vun**	**One copy for vun & multiple copies for gma**	**Multiple copies for both species**	**One copy for both species**	**Ungrouped proteins**
Numbers of families	1541	451	549	10196	4936	3912	
Numbers of proteins	6358	1859	2477	33763	22908	7824	10628
Numbers of gma proteins	6358		549	23567	13393	3912	8265
Percentage of gma proteins (%)	11.34		0.98	42.05	23.90	6.98	14.75
Numbers of vun proteins		1859	1928	10196	9515	3912	2363
Percentage of vun proteins (%)		6.24	6.48	34.25	31.96	13.14	7.94

Supplementary Table S2 | PIN1 interacting proteins with similar expression levels in *Glycine max* and *Vigna unguiculate*

Protein	Gene ID	Orthologs genes in soybean	Relative expression level	Orthologs genes in cowpea	Relative expression level
ABCB19	*AT3G28860*	*Glyma.13G063700*	0.241	*Vigun04g051400*	0.203
ABCB1	*AT2G36910*	*Glyma.19G184300*	0.218	*Vigun01g162000*	0.243
DL1	*AT5G42080*	*Glyma.08G023300*	0.756	*Vigun03g035600*	0.869
PGP1	*AT2G39290*	*Glyma.18G302100*	0.227	*Vigun05g000100*	0.200
TOPP4	*AT2G39840*	*Glyma.14G065200*	0.530	*Vigun08g152500*	0.587

Supplementary Table S3 | BLATST results of *Medtr4g126160* in soybean

Protein	Total score	E value	Per.Ident (%)	Acc. Len	Accession
GmCKX3b-2	819	0	75.33	522	XP_014625159.1
GmCKX3b-3	811	0	74.00	524	XP_006595335.1
GmCKX3b-1	671	0	63.15	527	XP_003534915.2

Supplementary Table S4 | *CKX* gene family in *Phaseolus vulgaris*

Gene name	Gene ID	Gene name	Gene ID
PvCKX1	*Phvul.001G128800*	*PvCKX5*	*Phvul.009G060200*
PvCKX3a-1	*Phvul.003G136400*	*PvCKX6-1*	*Phvul.005G034000*
PvCKX3a-2	*Phvul.009G231700*	*PvCKX6-2*	*Phvul.011G014000*
PvCKX3b-1	*Phvul.003G136500*	*PvCKX7-1*	*Phvul.001G038800*
PvCKX3b-2	*Phvul.009G231800*	*PvCKX7-2*	*Phvul.009G081800*

Supplementary Table S5 | Estimated time for segmental duplication events of *CKX* gene family in soybean

Segment pairs	Ks	Estimated time (mya)	Segment pairs	Ks	Estimated time (mya)
GmCKX1-2 & GmCKX6-2	2.033	167	*GmCKX1-1 & GmCKX1-2*	0.112	9
GmCKX3a-1 & GmCKX3a-2	0.885	73	*GmCKX3a-1 & GmCKX3a-3*	0.109	9
GmCKX3a-1 & GmCKX3a-4	0.889	73	*GmCKX3a-2 & GmCKX3a-4*	0.088	7
GmCKX3a-2 & GmCKX3a-3	0.908	74	*GmCKX5-1 & GmCKX5-2*	0.102	8
GmCKX3a-3 & GmCKX3a-4	0.876	72	*GmCKX6-1 & GmCKX6-3*	0.144	12
GmCKX7-1 & GmCKX7-2	0.857	70	*GmCKX7-2 & GmCKX7-3*	0.217	18
GmCKX7-1 & GmCKX7-3	0.892	73			

Supplementary Table S6 | Estimated time for segmental duplication event of *GmCKX6-2* segment

**Segment pairs**	**Ks**	**Estimated time (mya)**
Glyma.11G148900 & Glyma.19G136100	1.629	133
GmCKX6-2 & GmCKX1-2	2.033	167
Glyma.11G149400 & Glyma.19G134600	2.058	169
Glyma.11G149500 & Glyma.19G134500	NaN	NaN
Glyma.11G149900 & Glyma.19G133800	3.704	304
Glyma.11G151100 & Glyma.19G133000	1.904	156

Supplementary Table S7 | The interaction networks on *PIN1*, *CKX*, and SNPP-related genes in soybean and cowpea

Gene	Interacted gene	Confidence	Relative expression level	Homologies in *Arabidopsis*	References
**The interaction networks on *PIN1* in soybean and cowpea**					
GmPIN1	*Glyma.02g186700*	0.773	1.36	*AT2G41530* (*SFGH*)	Cummins et al. (2006)
(*Glyma.07G102500,*	*Glyma.03g183600*	0.632	0.40	*AT2G36910* (*ABCB1*)	
Glyma.08G054700,	*Glyma.05g171600*	0.976	NA	NA	
Glyma.09G176300)	*Glyma.10g106900*	0.773	0.80	*AT2G41530* (*SFGH*)	Cummins et al. (2006)
	Glyma.12g153700	0.605	NA	*AT1G30330*	
	Glyma.13g063700	0.815	0.24	*AT3G28860* (*ABCB19*)	
	Glyma.13g220100	0.976	0.19	*AT2G34650* (*PID*)	Lee et al. (2006)
	Glyma.14g217700	0.773	0.17	*AT1G19850*	
	Glyma.17g256500	0.773	0.16	*AT1G19850*	
	Glyma.19g021500	0.815	0.64	NA	
VuPIN1	*Vigun02g010200*	0.812	0.22	*AT1G50370*	
(*Vigun04g031500*)	*Vigun01g162000*	0.865	0.24	*AT2G36910* (*ABCB1*)	
	Vigun03g083000	0.884	NA	NA	
	Vigun03g192400	0.901	0.65	*AT1G23080* (*PIN7*)	
	Vigun03g181200	0.871	0.77	*AT2G43790* (*MPK6*)	Jia et al. (2016)

	Vigun04g051400	0.900	0.20	*AT3G28860* (*ABCB19*)	
	Vigun06g148700	0.887	2.25	*AT5G55910* (*D6PK*)	Zourelidou et al. (2014)
	Vigun02g031300	0.901	0.10	*AT1G23080* (*PIN7*)	
	Vigun06g179800	0.884	0.33	*AT2G34650* (*PID*)	Lee et al. (2006)
	Vigun07g072700	0.865	0.06	*AT2G36910* (*ABCB1*)	
**The interaction networks on *CKX* in soybean and cowpea**					
GmCKX3	*Glyma.12g079700*	0.642	0.56	*AT1G08110*	
(*Glyma.15G170300,*	*Glyma.06g109200*	0.641	0.60	*AT1G77760*	
Glyma.09G063900)	*Glyma.18g216400*	0.658	0.13	*AT4G23660*	
	Glyma.09g272700	0.658	NA	*AT4G23660*	
(*Glyma.17G054500*)	*Glyma.08g194400*	0.721	0.14	*AT5G49010* (*SLD5*)	Meinke. (2020)
,	*Glyma.01g004700*	0.676	NA	*AT4G29170*	
	Glyma.18g202800	0.672	0.51	*AT3G51840* (*ACX4*)	Rylott et al. (2003)
	Glyma.08g234900	0.726	NA	*AT1G16330*	
	Glyma.07g011200	0.721	NA	*AT5G49010* (*SLD5*)	Meinke. (2020)
VuCKX3	*Vigun07g098500*	0.558	0.29	NA	
(*Vigun09g033600,*	*Vigun10g174500*	0.543	0.20	*AT5G49010* (*SLD5*)	Meinke. (2020)
Vigun03g392300)	*Vigun10g081200*	0.555	1.98	*AT3G51840* (*ACX4*)	Rylott et al. (2003)
	Vigun03g323400	0.758	NA	*AT5G33320*	
	Vigun09g087400	0.758	1.34	*AT1G43310*	Wang et al. (2008)

VuCKX5	*Vigun11g190201*	0.588	NA	NA	
(*Vigun09g248900*)	*Vigun10g081200*	0.651	1.98	*AT3G51840* (*ACX4*)	Rylott et al. (2003)
	Vigun05g204700	0.666	NA	NA	
	Vigun02g198700	0.586	NA	*AT4G21200*	
	Vigun06g210000	0.612	0.12	NA	
**The interaction networks on SNPP-related in soybean and cowpea**					
GmCKX5-1	*Glyma.03g200800*	0.711	NA	*AT5G06300*	
(*Glyma.04g028900*)	*Glyma.12g086300*	0.734	NA	*AT2G27760 (IPT2)*	Miyawaki et al. (2004; 2006)
	Glyma.11g188100	0.734	0.16	*AT2G27760 (IPT2)*	Miyawaki et al. (2004; 2006)
	Glyma.13g271500	0.810	0.06	*AT5G20040 (IPT9)*	Miyawaki et al. (2004; 2006)
	Glyma.U017400	0.811	0.57	*AT5G36890*	
Glyma.04g136200	*Glyma.01g155300*	0.716	0.54	*AT5G65930*	
	Glyma.11g089400	0.716	0.08	*AT5G65930*	
	Glyma.09g194900	0.716	0.14	*AT5G65930*	
	Glyma.07g259400	0.846	NA	*AT4G21820*	Du et al. 2005; Midhat et al. (2018)
	Glyma.17g014900	0.846	NA	*AT4G21820*	Du et al. 2005; Midhat et al. (2018)
GmCKX5-2	*Glyma.03g200800*	0.711	NA	*AT5G06300*	
(Glyma.06g028900)	*Glyma.12g086300*	0.734	NA	*AT2G27760 (IPT2)*	Miyawaki et al. (2004; 2006)
	Glyma.11g188100	0.734	0.16	*AT2G27760 (IPT2)*	Miyawaki et al. (2004; 2006)

	Glyma.13g271500	0.810	0.06	*AT5G20040 (IPT9)*	Miyawaki et al. (2004; 2006)
	Glyma.U017400	0.811	0.57	*AT5G36890*	
	Glyma.08g054700	0.542	0.08	*AT1G73590*	
	Glyma.07g102500	0.542	0.17	*AT1G73590*	
Glyma.09g280200	*Glyma.04g214700*	0.644	0.05	*AT3G18690*	
	Glyma.04g239400	0.619	0.12	*AT3G18690*	
	Glyma.05g190000	0.644	NA	*AT3G18690*	
	Glyma.06g151400	0.644	0.07	*AT3G18690*	
	Glyma.08g147600	0.644	NA	*AT3G18690*	
Glyma.10g002200	*Glyma.01g155300*	0.716	0.54	*AT5G65930*	
	Glyma.07g259400	0.846	NA	*AT4G21820*	Du et al. (2005); Midhat et al. (2018)
	Glyma.09g194900	0.716	0.14	*AT5G65930*	
	Glyma.11g089400	0.716	0.08	*AT5G65930*	
	Glyma.17g014900	0.846	NA	*AT4G21820*	Du et al. (2005); Midhat et al. (2018)
Glyma.10g089300	*Glyma.03g159100*	0.905	4.61	*AT2G30570*	
	Glyma.09g250800	0.915	3.38	*AT4G12800*	
	Glyma.18g241700	0.915	8.42	*AT4G12800*	
	Glyma.12g092000	0.917	1.95	*AT1G08380*	

	Glyma.11g181100	0.917	3.83	*AT1G08380*	
Glyma.11g157100	*Glyma.02g250700*	0.735	NA	*AT3G55580*	
	Glyma.07g259400	0.846	NA	*AT4G21820*	Du et al. (2005); Midhat et al. (2018)
	Glyma.U034500	0.782	1.14	*AT5G60100*	
	Glyma.12g119200	0.739	0.68	*AT1G28480*	
	Glyma.17g014900	0.846	NA	*AT4G21820*	Du et al. (2005); Midhat et al. (2018)
Glyma.13g064700	*Glyma.07g024200*	0.772	7.86	*AT3G05610*	
	Glyma.07g024500	0.772	8.59	*AT3G05610*	
	Glyma.07g024600	0.772	8.23	*AT3G05610*	
	Glyma.U016300	0.806	NA	*AT4G35670*	
	Glyma.16g014100	0.804	0.30	*AT1G53830*	
	Glyma.13g060900	0.758	NA	*AT3G29090*	
Glyma.13g060900	*Glyma.04g246800*	0.822	0.34	*AT1G21690*	
	Glyma.06g116000	0.822	0.24	*AT1G21690*	
	Glyma.09g006000	0.797	NA	*AT1G11920*	
	Glyma.10g046100	0.832	NA	*AT3G55140*	
	Glyma.U016300	0.806	NA	*AT4G35670*	
Glyma.13g104700	*Glyma.11G20000*	0.642	NA	*AT5G58290*	

	Glyma.09g272700	0.658	NA	*AT4G23660*	
	Glyma.06g109200	0.641	0.06	*AT1G37130*	
	Glyma.12g079700	0.642	0.56	*AT1G08110*	
	Glyma.18g216400	0.658	0.13	*AT4G23660*	
Glyma.17g128900	*Glyma.01g155300*	0.716	0.54	*AT5G65930*	
	Glyma.07g259400	0.846	NA	*AT4G21820*	Du et al. (2005); Midhat et al. (2018)
	Glyma.09g194900	0.716	0.14	*AT5G65930*	
	Glyma.11g089400	0.716	0.08	*AT5G65930*	
	Glyma.17g014900	0.846	NA	*AT4G21820*	Du et al. (2005); Midhat et al. (2018)
LN	*Glyma.07g052100*	0.707	0.27	*AT2G46680*	
(*Glyma.20g116200*)	*Glyma.07g133700*	0.707	NA	*AT5G43290*	
	Glyma.20g184500	0.780	NA	*AT1G31310*	
	Glyma.03g194700	0.723	NA	*AT5G03840 (TFL1)*	Zhang et al. (2020)
	Glyma.19g194300	0.722	NA	*AT5G03840 (TFL1)*	Zhang et al. (2020)
	Glyma.07g102500	0.261	0.17	*AT1G73590*	
	Glyma.08g054700	0.261	0.08	*AT1G73590*	
The genes interacted with other SNPP-related genes in cowpea					
Vigun01g067000	*Vigun05g281200*	0.518	0.07	*AT1G15210*	

	Vigun07g171800	0.410	0.47	*AT3G23280*
	Vigun11g209900	0.995	0.10	*AT3G47450*
Vigun02g033000	*Vigun05g118000*	0.625	NA	*AT5G43290*
	Vigun09g099400	0.613	0.36	*AT5G50320*
	Vigun02g131100	0.849	NA	*AT2G18380*
	Vigun04g196200	0.635	NA	*AT3G27650*
Vigun03g314100	*Vigun05g281200*	0.518	0.07	*AT1G15210*
	Vigun07g117700	0.483	NA	*AT1G02920*
	Vigun06g220900	0.475	3.03	*AT3G57150*
	Vigun11g209900	0.993	0.10	*AT3G47450*
Vigun04g049700	*Vigun09g042900*	0.991	NA	*AT5G20860*
	Vigun03g199200	0.994	0.45	*AT2G43860*
	Vigun03g401000	0.988	0.06	NA
	Vigun07g128100	0.991	0.04	NA
	Vigun06g097800	0.991	NA	NA
Vigun04g049800	*Vigun09g042900*	0.991	NA	*AT5G20860*
	Vigun03g199200	0.994	0.45	*AT2G43860*
	Vigun03g401000	0.988	0.06	NA

	Vigun07g128100	0.991	0.04	NA	
	Vigun06g097800	0.991	NA	NA	
Vigun04g049900	*Vigun09g042900*	0.991	NA	*AT5G20860*	
	Vigun03g199200	0.994	0.45	*AT2G43860*	
	Vigun03g401000	0.988	0.06	NA	
	Vigun07g128100	0.991	0.04	NA	
	Vigun06g097800	0.991	NA	NA	
Vigun04g081900	*Vigun05g281200*	0.518	0.07	*AT1G15210*	
	Vigun07g171800	0.410	0.47	*AT3G23280*	
	Vigun11g209900	0.995	0.10	*AT3G47450*	
Vigun05g092400	*Vigun03g064800*	0.999	0.18	*AT3G18690*	
	Vigun09g154300	0.999	0.13	NA	
Vigun07g002900	*Vigun04g039000*	0.896	7.84	*AT1G65960*	
	Vigun05g040700	0.987	5.67	*AT3G29000*	Du et al. (2005); Midhat et al. (2018)
	Vigun03g412600	0.932	4.30	*AT5G49480 (CP1)*	Du et al. (2005); Midhat et al. (2018)
	Vigun01g098200	0.896	4.94	*AT1G66410 (CAM4)*	Du et al. (2005); Midhat et al. (2018)
	Vigun11g209900	0.997	0.10	*AT3G47450*	
VuLN	*Vigun05g118000*	0.625	NA	*AT5G43290*	

(*Vigun07g269900*)	*Vigun09g099400*	0.613	0.36	*AT5G50320*
	Vigun02g131100	0.849	NA	*AT2G18380*
	Vigun04g196200	0.635	NA	*AT3G27650*
Vigun09g102000	*Vigun05g281200*	0.518	0.07	*AT1G15210*
	Vigun07g117700	0.483	NA	*AT1G02920*
	Vigun06g220900	0.475	3.03	*AT3G57150*
	Vigun11g209900	0.993	0.10	*AT3G47450*
Vigun10g092300	*Vigun05g092400*	0.993	1.18	*AT2G30250*
	Vigun03g064800	0.932	0.18	*AT3G18690*
	Vigun09g154300	0.956	0.13	NA
Vigun11g216900	*Vigun11g124700*	0.929	7.65	*AT1G08380*
	Vigun09g257300	0.868	31.13	*AT1G20340*
	Vigun09g048600	0.922	6.07	*AT1G30380*
	Vigun01g004300	0.926	10.03	*AT1G55670*
	Vigun07g149400	0.882	2.02	*AT1G54780*

NA=Not Available

References

1. Cummins, I., McAuley, K., Fordham-Skelton, A., Schwoerer, R., Steel, P. G., Davis, B. G., et al. (2006). Unique regulation of the active site of the serine esterase S-formylglutathione hydrolase. *J. Mol. Biol*. 359, 422–432. doi:10.1016/j.jmb.2006.03.048

2. Du L, Poovaiah BW. (2005). Ca2+/calmodulin is critical for brassinosteroid biosynthesis and plant growth. *Nature* 437:741–745. doi:10.1038/nature03973
3. Jia, W., Li, B., Li, S., Liang, Y., Wu, X., Ma, M., et al. (2016). Mitogen-activated protein kinase cascade *MKK7-MPK6* plays important roles in plant development and regulates shoot branching by phosphorylating *PIN1* in *Arabidopsis*. *PLoS Biol.* 14, e1002550. doi: 10.1371/journal.pbio. 1002550
4. Lee, S. H., and Cho, H. T. (2006). *PINOID* positively regulates auxin efflux in *Arabidopsis* root hair cells and tobacco cells. *Plant Cell* 18, 1604–1616. doi: 10.1105/tpc.105.035972
5. Meinke, D. W. (2020). Genome-wide identification of EMBRYO-DEFECTIVE (EMB) genes required for growth and development in *Arabidopsis*. *New Phytol.* 226, 306–325. doi: 10.1111/nph.16071
6. Midhat U, Ting MKY, Teresinski HJ, Snedden WA. (2018). The calmodulin-like protein, *CML39*, is involved in regulating seed development, germination, and fruit development in *Arabidopsis. Plant Mol. Biol*. 96:375–392. doi:10.1007/s11103-018-0703-3
7. Miyawaki, K., Tarkowski, P., Matsumoto-Kitano, M., Kato, T., Sato, S., Tarkowska, D., et al. (2006). Roles of *Arabidopsis* ATP/ADP isopentenyltransferases and tRNA isopentenyltransferases in cytokinin biosynthesis. *Proc. Natl. Acad. Sci. U S A* 103, 16598–16603. doi:10.1073/pnas.0603522103
8. Miyawaki K, Matsumoto-Kitano M, Kakimoto T. (2004). Expression of cytokinin biosynthetic isopentenyltransferase genes in *Arabidopsis*: tissue specificity and regulation by auxin, cytokinin, and nitrate. *Plant J*. 37:128–138. doi:10.1046/j.1365-313x.2003.01945.x
9. Rylott, E. L., Rogers, C. A., Gilday, A. D., Edgell, T., Larson, T. R., and Graham, I. A. (2003). *Arabidopsis* mutants in short- and medium-chain acyl-CoA oxidase activities accumulate acyl-CoAs and reveal that fatty acid beta-oxidation is essential for embryo development. *J. Biol. Chem.* 278, 21370–21377. doi: 10.1074/jbc.M30082 6200\
10. Wang, Y., Zhang, W. Z., Song, L. F., Zou, J. J., Su, Z., and Wu, W. H. (2008). Transcriptome analyses show changes in gene expression to accompany pollen germination and tube growth in *Arabidopsis*. *Plant Physiol.* 148, 1201–1211. doi: 10.1104/pp.108.126375
11. Zhang, B., Li, C., Li, Y., & Yu, H. (2020). Mobile TERMINAL FLOWER1 determines seed size in *Arabidopsis. Nature Plants* 6, 1146–1157. doi:10.1038/s41477-020-0749-5

12. Zourelidou, M., Absmanner, B., Weller, B., Barbosa, I. C., Willige, B. C., Fastner, A., et al. (2014). Auxin efflux by PIN-FORMED proteins is activated by two different protein kinases, D6 PROTEIN KINASE and PINOID. *eLife* 3. e02860. doi: 10.7554/eLife.02860

Supplementary Table S8 | Prediction of miRNAs targeting calmodulin and calcium-binding proteins in soybean and cowpea with expected value of 3.0

Gene	miRNA	Expected	miRNA			Targeted gene			Inhibition
			Start	End	Aligned fragment	Start	End	Aligned fragment	
Glyma.17G014900	*gma-miR4405*	2.0	1	24	AUUCUAAGACGGUUAUCUGGGACC	4475	4498	CUGUUCAGAUAAUUGUCUUGGAGU	Cleavage
Glyma.07G259400	*gma-miR4405*	2.0	1	24	AUUCUAAGACGGUUAUCUGGGACC	4599	4622	CUGUACAGAUAAUUGUCUUGGAGU	Cleavage
Vigun03g412600	*016048_minus*	2.5	1	19	UUUUUUUUUUUUUUUUUUU	62	80	GAAAAAGAAAAAAGAAAGG	Cleavage

Supplementary Table S9 | The interaction networks on yield-related genes in soybean and cowpea

Known Gene	Traits	Interacted gene	Confidence	Relative expression level	Homologies in *Arabidopsis*	References
**The interaction networks on yield-related genes in soybean**						
PD1 (*Glyma.01G240100*)	Yield/Stress resistance	*Glyma.01G217500*	0.556	NA	*AT5G02320*	
		Glyma.02G019000	0.551	1.09	*AT1G14340*	
		Glyma.11G135300	0.553	0.07	*AT4G01570*	
		Glyma.18G142500	0.582	1.07	*AT1G10760*	
		Glyma.08G283700	0.582	4.49	*AT1G10760*	
CTP (*Glyma.05G022400*)	Yield/Flowering	*Glyma.02G253200*	0.605	0.66	*AT2G39890*	
		Glyma.11G226000	0.605	3.59	*AT2G39890*	
		Glyma.05G02781	0.613	NA	NA	
		Glyma.05G043100	0.613	0.07	*AT2G39890*	
		Glyma.17G125600	0.613	NA	*AT2G39890*	
PP2C-1 (*Glyma.17G221100*)	Yield (seed weight)/Domestication	*Glyma.02G251100*	0.705	0.21	*AT3G55610*	
		Glyma.03G261000	0.607	0.26	*AT1G01090*	
		Glyma.14G065600	0.705	0.57	*AT3G55610*	
		Glyma.19G260000	0.702	0.10	*AT1G01090*	

		Glyma.18G034300	0.705	0.24	*AT3G55610*	
GmCIF1 (*Glyma.17G036300*)	Yield (seed weight)	*Glyma.02G075200*	0.605	NA	*AT1G71850*	
		Glyma.03G200500	0.603	0.35	*AT3G52520*	
		Glyma.04G228400	0.581	0.52	*AT5G24490*	
		Glyma.16G157200	0.605	NA	*AT1G71850*	
		Glyma.19G198100	0.603	0.15	*AT3G52520*	
GA20OX (*Glyma.07G081700*)	Yield (seed size/weight)/ Seed quality (oil content)/Domestication	*Glyma.01G199800*	0.900	NA	*AT2G32440* (*KAO2*)	Helliwell et al. (2001); Regnault et al. (2014)
		Glyma.04G033000	0.837	NA	*AT2G18500*	
		Glyma.U002000	0.875	NA	*AT1G47990* (*GA2OX4*)	Jasinski et al. (2005)
		Glyma.15G135200	0.900	0.11	*AT2G32440* (*KAO2*)	Helliwell et al. (2001); Regnault et al. (2014)
		Glyma.09G029400	0.900	0.18	*AT1G05160* (*CYP88A3*)	Helliwell et al. (2001); Regnault et al. (2014)
NFYA (*Glyma.02G303800*)	Yield (seed size/weight)/ Seed quality (oil content)/Domestication	*Glyma.07G268100*	0.787	NA	*AT5G47670*	
		Glyma.11G148000	0.797	0.05	*AT1G07980*	
		Glyma.12G069100	0.797	0.14	*AT1G07980*	

		Glyma.08G165700	0.820	0.65	*AT1G56170*
		Glyma.15G261300	0.820	1.04	*AT1G56170*
SoyWRKY15a (*Glyma.05G096500*)	Yield (seed size)/Domestication	*Glyma.02G204500*	0.597	NA	*AT5G57920*
		Glyma.07G039400	0.596	NA	*AT3G13840*
		Glyma.08G214000	0.599	0.25	*AT1G15290*
		Glyma.11G189500	0.599	0.43	*AT5G22650*
		Glyma.16G008200	0.596	NA	*AT3G13840*
GmCYP78A70 (*Glyma.01G061100*)	Yield (seed size)	*Glyma.13G206600*	0.572	0.08	*AT4G21770*
		Glyma.20G156700	0.574	NA	*AT5G07280*
		Glyma.10G237900	0.574	0.06	*AT5G07280*
		Glyma.03G226800	0.606	6.75	*AT3G14610*
		Glyma.07G274000	0.616	0.16	*AT3G59900*
GmCYP78A57 (*Glyma.02G119600*)	Yield (seed size)	*Glyma.02G103800*	0.721	1.06	*AT4G34100*
		Glyma.02G106500	0.724	NA	*AT1G26830*
		Glyma.02G111000	0.724	NA	*AT1G26690*
		Glyma.04G107400	0.734	0.80	*AT2G36070*
		Glyma.17G123100	0.788	NA	*AT1G68780*
GmCYP78A72 (*Glyma.19G240800*)	Yield (seed size)	*Glyma.07G274000*	0.616	0.16	*AT3G59900*

		Glyma.10G237900	0.574	0.06	*AT5G07280*	
		Glyma.13G206600	0.572	0.08	*AT4G21770*	
		Glyma.15G106100	0.572	0.09	*AT4G21770*	
		Glyma.20G156700	0.574	NA	*AT5G07280*	
GmKIX8-1 (*Glyma.17G112800*)	Yield (seed size)	*Glyma.04G071400*	0.725	0.18	*AT4G32570*	
		Glyma.06G072700	0.725	0.32	*AT4G32570*	
		Glyma.10G244400	0.696	0.48	*AT4G14720*	
		Glyma.20G150000	0.696	0.43	*AT4G14720*	
		Glyma.17G205200	0.725	NA	*AT4G32570*	
BIG SEEDS1 (*Glyma.10G244400*)	Yield (seed size)	*Glyma.01G146400*	0.951	1.29	*AT4G28910*	
		Glyma.04G071400	0.940	0.18	*AT4G32570*	
		Glyma.06G072700	0.940	0.32	*AT4G32570*	
		Glyma.09G193900	0.951	1.19	*AT4G28910*	
		Glyma.17G205200	0.940	NA	*AT4G32570*	
GmCYP78A10 (*Glyma.05G019200*)	Yield (seed size)	*Glyma.02G057500*	0.652	NA	*AT3G50660* (*DWF4*)	Fujiyama et al. (2019)
		Glyma.02G256800	0.637	NA	*AT5G05690*	
		Glyma.03G226800	0.605	6.75	*AT3G14610*	
		Glyma.07G274000	0.616	0.16	*AT3G59900*	

		Glyma.11G067700	0.647	0.37	*AT3G50660* (*DWF4*)	Fujiyama et al. (2019)
GmMYB14 (*Glyma.19G164600*)	Yield (plant architecture)/Stress resistance (drought)	*Glyma.06G295700*	0.705	3.55	*AT5G54160*	
		Glyma.12G109800	0.705	1.96	*AT5G54160*	
		Glyma.12G201500	0.648	0.54	*AT2G20810*	
		Glyma.13G300700	0.648	0.22	*AT2G20810*	
		Glyma.U020100	0.639	0.70	*AT5G60410*	
Dt1 (*Glyma.19G194300*)	Yield (plant architecture)/Domestication	*Glyma.09G111600*	0.940	NA	*ATCG00790*	
		Glyma.11G163000	0.952	0.15	*AT1G16870*	
		Glyma.13G166400	0.932	0.82	*AT5G20180*	
		Glyma.18G056300	0.952	0.11	*AT1G16870*	
		Glyma.19G07400	0.940	NA	NA	
Dt2 (*Glyma.18G273600*)	Yield (plant architecture)	*Glyma.08G255200*	0.610	0.05	*AT3G02380*	
		Glyma.03G194700	0.729	NA	*AT5G03840*	
		Glyma.03G263200	0.606	0.22	*AT1G72320*	
		Glyma.10G047900	0.608	1.34	*AT5G02800*	
		Glyma.19G194300	0.729	NA	*AT5G03840*	
GmSPL9a (*Glyma.02G177500*)	Yield (plant architecture)	*Glyma.09G229900*	0.567	0.22	*AT5G61460*	

		Glyma.19G178200	0.580	0.32	*AT2G28550*
		Glyma.03G177500	0.580	0.12	*AT2G28550*
		Glyma.20G208800	0.703	0.96	*AT4G14340*
		Glyma.10G181700	0.703	1.08	*AT4G14340*
GmSPL9b (*Glyma.09G113800*)	Yield (plant architecture)	*Glyma.03G177500*	0.580	0.12	*AT2G28550*
		Glyma.09G229900	0.567	0.22	*AT5G61460*
		Glyma.10G181700	0.703	1.08	*AT4G14340*
		Glyma.19G178200	0.580	0.32	*AT2G28550*
		Glyma.20G208800	0.703	0.96	*AT4G14340*
GmSPL9c (*Glyma.03G143100*)	Yield (plant architecture)	*Glyma.09G229900*	0.567	0.22	*AT5G61460*
		Glyma.19G178200	0.580	0.32	*AT2G28550*
		Glyma.03G177500	0.580	0.12	*AT2G28550*
		Glyma.10G181700	0.703	1.08	*AT4G14340*
		Glyma.20G208800	0.703	0.96	*AT4G14340*
GmSPL9d (*Glyma.19G146000*)	Yield (plant architecture)	*Glyma.04G085000*	0.639	0.12	*AT4G32880*
		Glyma.04G125700	0.628	NA	*AT5G06100*
		Glyma.05G001600	0.591	0.77	*AT3G23590*
		Glyma.10G181700	0.703	1.08	*AT4G14340*

		Glyma.20G208800	0.703	0.96	*AT4G14340*
GmILPA1 (*Glyma.11G026400*)	Yield (petiole angle)	*Glyma.02G45391*	0.994	NA	NA
		Glyma.06G228700	0.992	0.29	*AT1G78770*
		Glyma.07G190600	0.999	0.39	*AT4G21530*
		Glyma.12G156300	0.992	0.07	*AT1G78770*
		Glyma.14G030500	0.994	0.10	*AT1G06590*
CRY2a (*Glyma.20G209900*)	Yield (leaf senescence)	*Glyma.02G267800*	0.987	0.18	*AT2G32950*
		Glyma.04G012500	0.970	NA	*AT4G34530*
		Glyma.07G058200	0.986	1.45	*AT2G46340*
		Glyma.14G049700	0.987	0.31	*AT2G32950*
		Glyma.16G027200	0.986	1.56	*AT2G46340*
CIB1 (*Glyma.11G117100*)	Yield (leaf senescence)	*Glyma.04G101500*	0.915	0.61	*AT5G13630*
		Glyma.20G209900	0.970	2.87	*AT1G08520*
		Glyma.10G180600	0.970	3.69	*AT4G25080*
		Glyma.02G005700	0.970	0.16	*AT4G25080*
		Glyma.18G07820	0.970	NA	*AT1G08520*
GmCHLI1 (*Glyma.13G232500*)	Yield (chlorophyll degradation)	*Glyma.03G137000*	0.990	0.52	*AT5G13630*
		Glyma.01G226700	0.992	0.17	*AT1G08520*

		Glyma.05G200500	0.977	0.18	*AT4G25080*
		Glyma.08G007900	0.977	NA	*AT4G25080*
		Glyma.11G016000	0.992	0.21	*AT1G08520*
GmCHLI2 (*Glyma.15G080200*)	Yield (chlorophyll degradation)	*Glyma.03G137000*	0.976	0.52	*AT5G13630*
		Glyma.01G226700	0.992	0.17	*AT1G08520*
		Glyma.05G200500	0.977	0.18	*AT4G25080*
		Glyma.08G007900	0.977	NA	*AT4G25080*
		Glyma.11G016000	0.992	0.21	*AT1G08520*
GmCHLI3 (*Glyma.07G204300*)	Yield (chlorophyll degradation)	*Glyma.03G137000*	0.986	0.52	*AT5G13630*
		Glyma.01G226700	0.992	0.17	*AT1G08520*
		Glyma.05G200500	0.983	0.18	*AT4G25080*
		Glyma.08G007900	0.983	NA	*AT4G25080*
		Glyma.11G016000	0.992	0.21	*AT1G08520*
GmCHLI4 (*Glyma.13G171800*)	Yield (chlorophyll degradation)	*Glyma.03G137000*	0.976	0.52	*AT5G13630*
		Glyma.01G226700	0.992	0.17	*AT1G08520*
		Glyma.05G200500	0.977	0.18	*AT4G25080*
		Glyma.08G007900	0.977	NA	*AT4G25080*
		Glyma.11G016000	0.992	0.21	*AT1G08520*

D2 (*Glyma.11G027400*)	Yield (chlorophyll degradation)	*GLYMA02G45391*	0.994	NA	NA
		Glyma.06G228700	0.992	0.29	*AT1G78770*
		Glyma.07G190600	0.999	0.39	*AT4G21530*
		Glyma.12G156300	0.992	0.07	*AT1G78770*
		Glyma.14G030500	0.994	0.10	*AT1G06590*
D1 (*Glyma.01G214600*)		*Glyma.07G085700*	0.860	4.26	*AT4G13250*
		Glyma.09G226000	0.849	0.25	*AT5G13800*
		Glyma.10G003200	0.898	0.94	*AT5G43860*
		Glyma.14G012500	0.859	0.84	*AT4G37000*
		Glyma.09G191200	0.860	6.84	*AT4G13250*
GmPSKγ1 (*Glyma.02G126200*)	Yield	NA			
PS (*Glyma.12G187200*)	Yield/Stress resistance	NA			
P1 (*Glyma.09G278000*)	Yield/Stress resistance	NA			

The interaction networks on yield-related genes in cowpea

Gene	Homology in soybean	Interacted gene	Confidence	Relative expression level	Homologies in *Arabidopsis*	References
Vigun09g057900	*GmKIX8-1* (*Glyma.17G112800*)	*Vigun05g297000*	0.853	0.68	*AT1G15750* (*TPL*)	Liscum et al. (2002)
		Vigun09g204700	0.895	0.18	*AT4G32570*	
		Vigun07g235400	0.933	0.24	*AT4G14713*	
		Vigun04g009800	0.910	0.95	*AT4G28910*	
Vigun08g214100	*NFYA* (*Glyma.02G303800*)	*Vigun01g181100*	0.990	0.43	*AT3G05690*	
		Vigun05g009500	0.999	NA	NA	
		Vigun01g054400	0.998	0.26	*AT1G56170*	
		Vigun09g105500	0.991	0.33	*AT3G48590*	
		Vigun06g164900	0.998	NA	NA	
Vigun03g280900	*SoyWRKY15a* (*Glyma.05G096500*)	*Vigun04g132000*	0.805	0.27	*AT1G21200*	
		Vigun03g160400	0.798	0.35	*AT1G14740*	
		Vigun07g217400	0.805	0.47	*AT1G21200*	
		Vigun02g015900	0.798	0.95	*AT1G14740*	
		Vigun11g214100	0.798	0.51	*AT3G63500*	
Vigun07g027100	*GmSPL9a* (*Glyma.02G177500*)	*Vigun01g013100*	0.401	0.11	*AT3G11440*	
	GmSPL9b (*Glyma.09G113800*)	*Vigun07g172300*	0.415	0.72	*AT1G04440*	

	GmSPL9c (*Glyma.03G143100*)	*Vigun06g194600*	0.597	NA	NA	
	GmSPL9d (*Glyma.19G146000*)	*Vigun01g232900*	0.584	0.09	*AT1G10280*	
		Vigun09g191500	0.487	0.46	*AT1G52150*	
Vigun06g194100	*GmCHLI1* (*Glyma.13G232500*)	*Vigun07g131800*	0.974	0.37	*AT4G01690*	
	GmCHLI2 (*Glyma.15G080200*)	*Vigun04g034300*	0.968	0.72	*AT5G26030*	
	GmCHLI3 (*Glyma.07G204300*)	*Vigun01g115300*	0.999	0.82	*AT5G13630*	
	GmCHLI4 (*Glyma.13G171800*)	*Vigun03g052400*	0.977	1.07	*AT4G25080*	
		Vigun02g183600	0.989	0.28	*AT1G08520*	
Vigun02g198000	*PD1* (*Glyma.01G240100*)	*Vigun01g056400*	0.755	0.96	*AT1G79430*	
		Vigun09g218400	0.431	1.95	*AT2G26250*	
		Vigun11g035600	0.755	0.70	*AT1G79430*	
		Vigun04g096900	0.792	7.22	*AT1G67090*	
Vigun03g411300	*GmCIF1* (*Glyma.17G036300*)	*Vigun07g062400*	0.834	0.99	*AT2G36190* (*CWIN4*)	Liao et al. (2020)
		Vigun01g175600	0.834	0.04	*AT2G36190* (*CWIN4*)	Liao et al. (2020)
Vigun10g116900	*GA20OX* (*Glyma.07G081700*)	*Vigun05g288700*	0.999	0.26	*AT1G15550*	

		Vigun10g047000	0.999	NA	NA
		Vigun02g198700	0.999	NA	*AT4G21200*
		Vigun11g190200	0.999	NA	NA
		Vigun05g204700	0.999	NA	NA
Vigun03g340500	*CTP* (*Glyma.05G022400*)	*Vigun03g318000*	0.402	0.05	NA
Vigun08g046900	*PP2C-1* (*Glyma.17G221100*)	*Vigun05g290300*	0.404	0.03	*AT4G13560*
		Vigun03g209400	0.459	0.43	*AT1G26830*
		Vigun03g349200	0.555	0.18	*AT3G18520*
		Vigun02g045800	0.555	0.22	*AT1G69180*
		Vigun03g189600	0.505	0.88	*AT4G34100*
Vigun06g069600	*NFYA* (*Glyma.02G303800*)	*Vigun01g181100*	0.990	0.43	*AT3G05690*
		Vigun05g009500	0.999	NA	NA
		Vigun01g054400	0.998	0.26	*AT1G56170*
		Vigun09g105500	0.991	0.33	*AT3G48590*
		Vigun06g164900	0.998	NA	NA
Vigun02g146400	*SoyWRKY15a* (*Glyma.05G096500*)	*Vigun04g132000*	0.805	0.27	*AT1G21200*
		Vigun03g160400	0.798	0.35	*AT1G14740*
		Vigun07g217400	0.805	0.47	*AT1G21200*

		Vigun02g015900	0.798	0.95	*AT1G14740*	
		Vigun11g214100	0.798	0.51	NA	
Vigun02g047800	*GmCYP78A70* (*Glyma.01G061100*)	*Vigun11g215700*	0.501	0.57	NA	
	GmCYP78A57 (*Glyma.02G119600*)	*Vigun09g179600*	0.582	NA	NA	
	GmCYP78A72 (*Glyma.19G240800*)	*Vigun03g350200*	0.686	0.23	*AT2G26910*	
		Vigun02g001300	0.557	0.07	*AT2G33560*	
		Vigun02g115000	0.532	0.23	*AT1G20590*	
Vigun03g358000	*GmKIX8-1* (*Glyma.17G112800*)	*Vigun05g297000*	0.853	0.68	*AT1G15750* (*TPL*)	Liscum et al. (2002)
		Vigun09g204700	0.895	0.18	*AT4G32570*	
		Vigun07g235400	0.933	0.24	*AT4G14713*	
		Vigun04g009800	0.910	0.95	*AT4G28910*	
Vigun07g235400	*BIG SEEDS1* (*Glyma.10G244400*)	*Vigun09g057900*	0.933	0.25	NA	
		Vigun09g204700	0.890	0.18	*AT4G32570*	
		Vigun08g215000	0.931	NA	*AT5G35770*	
		Vigun09g204700	0.889	0.18	*AT4G32570*	
		Vigun04g009800	0.960	0.95	*AT4G28910*	
Vigun03g343100	*GmCYP78A10* (*Glyma.05G019200*)	*Vigun06g114600*	0.442	0.21	*AT5G28640* (*AN3*)	Meng et al. (2016)

		Vigun07g156800	0.442	0.51	*AT5G28640* (*AN3*)	Meng et al. (2016)
		Vigun02g089000	0.426	0.11	*AT5G10560*	
		Vigun02g001300	0.518	0.07	*AT2G33560*	
		Vigun10g150400	0.428	0.05	*AT1G01180*	
Vigun01g142900	*GmMYB14* (*Glyma.19G164600*)	*Vigun05g063400*	0.758	0.15	*AT2G46020*	
		Vigun05g280100	0.755	1.50	*AT1G51950* (*IAA18*)	Liscum et al. (2002)
		Vigun07g287100	0.755	3.26	*AT2G04630*	
		Vigun07g265600	0.801	NA	*AT1G15050* (*IAA34*)	Liscum et al. (2002)
		Vigun10g131000	0.758	0.22	*AT2G46020*	
Vigun01g173000	*Dt1* (*Glyma.19G194300*)	*Vigun05g004800*	0.764	16.24	*AT1G35160* (*GF14 PHI*)	Gampala et al. (2007); Keicher et al. (2017)
		Vigun09g255700	0.865	0.06	NA	
		Vigun09g178000	0.764	10.14	*AT1G78300* (*GRF2*)	Gampala et al. (2007); Keicher et al. (2017)
		Vigun07g059500	0.793	0.08	*AT3G10080*	
		Vigun02g119600	0.865	NA	*AT2G17770*	
Vigun05g029200	*Dt2* (*Glyma.18G273600*)	*Vigun09g021400*	0.513	NA	*AT1G78635*	
		Vigun01g173000	0.490	NA	*AT2G27550*	
		Vigun01g137700	0.835	NA	NA	
		Vigun07g106300	0.835	NA	NA	

		Vigun02g108700	0.506	0.17	*AT4G19190*
Vigun01g122900	*GmSPL9a* (*Glyma.02G177500*)	*Vigun01g013100*	0.401	0.11	*AT3G11440*
	GmSPL9b (*Glyma.09G113800*)	*Vigun07g172300*	0.415	0.72	*AT1G04440*
	GmSPL9c (*Glyma.03G143100*)	*Vigun06g194600*	0.597	NA	NA
	GmSPL9d (*Glyma.19G146000*)	*Vigun01g232900*	0.584	0.09	*AT1G10280*
		Vigun09g191500	0.487	0.46	*AT1G52150*
Vigun02g173100	*GmILPA1* (*Glyma.11G026400*)	*Vigun01g219000*	0.999	0.14	*AT2G04660*
		Vigun01g217700	0.999	0.07	*AT5G13840*
		Vigun01g145100	0.999	0.38	*AT1G04020*
		Vigun08g162100	0.999	0.02	*AT5G05560*
		Vigun03g114900	0.999	0.04	*AT4G21530*
Vigun07g171300	*CRY2a* (*Glyma.20G209900*)	*Vigun09g227400*	0.995	0.16	*AT2G25930*
		Vigun08g035200	0.995	0.60	*AT2G25930*
		Vigun10g142900	0.999	0.24	NA
		Vigun10g143000	0.999	0.21	*AT2G46340*
		Vigun10g177000	0.996	0.09	NA
Vigun11g176000	*CIB1*	*Vigun03g412400*	0.838	0.75	*AT3G06590*

	(*Glyma.11G117100*)				
		Vigun07g171300	0.890	3.10	*AT1G04400*
		Vigun06g130800	0.838	0.35	NA
		Vigun02g038500	0.870	0.37	*AT1G26260*
		Vigun07g008300	0.890	0.42	*AT3G06590*
Vigun03g173900	*GmCHLI1* (*Glyma.13G232500*)	*Vigun07g131800*	0.974	0.37	*AT4G01690*
	GmCHLI2 (*Glyma.15G080200*)	*Vigun04g034300*	0.968	0.72	*AT5G26030*
	GmCHLI3 (*Glyma.07G204300*)	*Vigun01g115300*	0.999	0.82	*AT5G13630*
	GmCHLI4 (*Glyma.13G171800*)	*Vigun03g052400*	0.980	1.07	*AT4G25080*
		Vigun02g183600	0.989	0.28	*AT1G08520*
Vigun02g171900	*D2* (*Glyma.11G027400*)	*Vigun05g098600*	0.999	0.42	*AT3G51820*
	D1 (*Glyma.01G214600*)	*Vigun07g003700*	0.990	0.83	*AT5G43860*
		Vigun07g049700	0.999	0.69	*AT1G04620*
		Vigun06g175000	0.999	0.71	*AT5G18660*
		Vigun09g158500	0.999	0.09	*AT1G44000*
Vigun05g089700	*P1* (*Glyma.09G278000*)	NA			

Vigun05g089800	*P1* (*Glyma.09G278000*)	NA
Vigun06g202800	*P1* (*Glyma.09G278000*)	NA

References

1. Fujiyama, K., Hino, T., Kanadani, M., Watanabe, B., Jae Lee, H., Mizutani, M., et al. (2019). Structural insights into a key step of brassinosteroid biosynthesis and its inhibition. *Nat Plant.* 5, 589–594. doi:10.1038/s41477-019-0436-6
2. Gampala, S. S., Kim, T. W., He, J. X., Tang, W., Deng, Z., Bai, M. Y., et al. (2007). An essential role for 14-3-3 proteins in brassinosteroid signal transduction in *Arabidopsis*. *Dev. Cell* 13, 177–189. doi:10.1016/j.devcel.2007.06.009
3. Helliwell, C. A., Chandler, P. M., Poole, A., Dennis, E. S., and Peacock, W. J. (2001). The CYP88A cytochrome P450, ent-kaurenoic acid oxidase, catalyzes three steps of the gibberellin biosynthesis pathway. *Proc. Natl. Acad. Sci. U S A* 98, 2065–2070. doi:10.1073/pnas.041588998
4. Jasinski, S., Piazza, P., Craft, J., Hay, A., Woolley, L., Rieu, I., et al. (2005). *KNOX* action in *Arabidopsis* is mediated by coordinate regulation of cytokinin and gibberellin activities. *Curr. Biol*. 15, 1560–1565. doi:10.1016/j.cub.2005.07.023
5. Keicher, J., Jaspert, N., Weckermann, K., Möller, C., Throm, C., Kintzi, A., et al. (2017). *Arabidopsis* 14-3-3 epsilon members contribute to polarity of *PIN* auxin carrier and auxin transport-related development. eLife, 6, e24336. doi:10.7554/eLife.24336
6. Liao, S., Wang, L., Li, J., & Ruan, Y. L. (2020). Cell wall invertase is essential for ovule development through sugar signaling rather than provision of carbon nutrients. *Plant Physiol.* 183, 1126–1144. doi:10.1104/pp.20.00400
7. Liscum, E., and Reed, J. W. (2002). Genetics of Aux/IAA and ARF action in plant growth and development. *Plant Mol. Biol*. 49, 387–400.
8. Meng, L. S., Wang, Y. B., Loake, G. J., and Jiang, J. H. (2016). Seed embryo development is regulated via an *AN3-MINI3* gene cascade. *Front. Plant Sci.* 7, 1645. doi:10.3389/fpls.2016.01645

9. Regnault, T., Davière, J. M., Heintz, D., Lange, T., and Achard, P. (2014). The gibberellin biosynthetic genes *AtKAO1* and *AtKAO2* have overlapping roles throughout *Arabidopsis* development. *Plant J.* 80, 462–474. doi:/10.1111/tpj.12648

Supplementary Table S10 | The expression levels (FPKM) of *CYP78A* family genes at different seed development stages in soybean and cowpea

Soybean gene	Gene expressional level (FPKM)				Homology in Cowpea	Gene expressional level (FPKM)			
	**12-14 DAF**	**22-24 DAF**	**5-6 mgWS**	**100-200 mgCOT**		**seed 8D**	**seed 10D**	**seed 14D**	**seed 18D**
GmCYP78A70 (*Glyma.01g061100*)	0.97	0.29	14.72	31.18					
GmCYP78A57 (*Glyma.02g119600*)	11.49	0.30	258.59	413.89	*Vigun02g047800*	22.14	10.06	0.44	0.00
GmCYP78A72 (*Glyma.19g240800*)	10.73	0.15	211.20	250.64					
GmCYP78A10 (*Glyma.05g019200*)	0.67	0.37	0.66	0.07	*Vigun03g343100*	0.87	0.34	0.12	0.01

DAF: Day After Flowering; WS: Whole Seed; COT: Cotyledon; D: Day.

Supplementary Table S11 | Cytokinin content in different tissues in soybean and cowpea

Case	Soybean				References	Cowpea			References
	Root	Leaf	Flower	Pod		Root	Shoot	Seed	
Original data	15 poml/g	15 poml/g	11 poml/g	170 poml/g	Le et al. (2012)	0.47 µg/g	2.3 mg/10g	90 µg/g	Thiagarajan et al. (1994)<br>El-Saeid et al. (2010)<br>Al-Amri (2018)
Transformed data	3.4e-3 µg/g	3.4e-3 µg/g	2.5e-3 µg/g	3.8e-2 µg/g		0.47µg/g	230 µg/g	90 µg/g	

References

1. Al-Amri, S. M. (2018). Functional activity of some growth regulators on yield components and endogenous hormones of cowpea plants (*Vigna sinensis* L.). *Agricultural Sciences* 9, 1229
2. El-Saeid, H. M., Abou-Hussein, S. D., and El-Tohamy, W. A. (2010). Growth characters, yield and endogenous hormones of cowpea plants in response to IAA application. *Research Journal of Agriculture and Biological Sciences* 6, 27-31
3. Le, D. T., Nishiyama, R., Watanabe, Y., Vankova, R., Tanaka, M., Seki, M., et al. (2012). Identification and expression analysis of cytokinin metabolic genes in soybean under normal and drought conditions in relation to cytokinin levels. P*LoS One* 7, e42411. https://doi.org/10.1371/journal.pone.0042411
4. Thiagarajan, T. R., and M. H. Ahmad. (1994). Phosphatase activity and cytokinin content in cowpeas (*Vigna unguiculata*) inoculated with a vesicular-arbuscular mycorrhizal fungus. *Biology and Fertility of Soils* 17 : 51-56
